# Bistability in oxidative stress response determines the migration behavior of phytoplankton in turbulence

**DOI:** 10.1073/pnas.2005944118

**Published:** 2021-01-25

**Authors:** Francesco Carrara, Anupam Sengupta, Lars Behrendt, Assaf Vardi, Roman Stocker

**Affiliations:** ^a^Institute for Environmental Engineering, Department of Civil, Environmental and Geomatic Engineering, ETH Zurich, 8093 Zurich, Switzerland;; ^b^Physics of Living Matter, Department of Physics and Materials Science, University of Luxembourg, 1511 Luxembourg City, Grand Duchy of Luxembourg;; ^c^Science for Life Laboratory, Department of Environmental Toxicology, Uppsala University, 75236 Uppsala, Sweden;; ^d^Department of Plant and Environmental Sciences, Weizmann Institute of Science, 7610001 Rehovot, Israel

**Keywords:** ROS, motility, photophysiology, harmful-algal-bloom, intermittency

## Abstract

Turbulence is an important determinant of phytoplankton physiology, often leading to cell stress and damage. Turbulence affects phytoplankton migration both by transporting cells and by triggering switches in migratory behavior, whereby vertically migrating cells can actively invert their direction of migration upon exposure to turbulent cues. However, a mechanistic link between single-cell physiology and vertical migration of phytoplankton in turbulence is currently missing. Here, by combining physiological and behavioral experiments with a mathematical model of stress accumulation and dissipation, we show that the mechanism responsible for the switch in the direction of migration in the marine raphidophyte *Heterosigma akashiwo* is the integration of reactive oxygen species (ROS) signaling generated by turbulent cues. Within timescales as short as tens of seconds, the emergent downward-migrating subpopulation exhibited a twofold increase in ROS, an indicator of stress, 15% lower photosynthetic efficiency, and 35% lower growth rate over multiple generations compared to the upward-migrating subpopulation. The origin of the behavioral split as a result of a bistable oxidative stress response is corroborated by the observation that exposure of cells to exogenous stressors (H_2_O_2_, UV-A radiation, or high irradiance), in lieu of turbulence, caused comparable ROS accumulation and an equivalent split into the two subpopulations. By providing a mechanistic link between the single-cell mechanics of swimming and physiology on the one side and the emergent population-scale migratory response and impact on fitness on the other, the ROS-mediated early warning response we discovered contributes to our understanding of phytoplankton community composition in future ocean conditions.

## Significance

Turbulence has long been known to drive phytoplankton fitness and species succession: motile species dominate in calmer environments and non-motile species in turbulent conditions. Yet a mechanistic understanding of the effect of turbulence on phytoplankton migratory behavior and physiology is lacking. By combining a method to generate turbulent cues, quantification of stress accumulation and physiology, and a mathematical model of stress dynamics, we show that motile phytoplankton use their mechanical stability to sense the intensity of turbulent cues and integrate these cues in time via stress signaling to trigger switches in migratory behavior. The stress-mediated warning strategy we discovered provides a paradigm for how phytoplankton cope with turbulence, thereby potentially governing which species will be successful in a changing ocean.

Turbulence regulates the distribution of dissolved and particulate matter in the ocean (1), and, along with light and nutrient supply (2), shapes the fluid dynamical (3) and evolutionary niches of phytoplankton in marine ecosystems (4) by selecting fundamental traits such as body size and shape (5), life history strategies, and motility characteristics (6). Larger cells such as diatoms benefit from turbulence through enhanced nutrient uptake (5–7), whereas turbulence is often detrimental for smaller motile phytoplankton, causing physiological impairment and physical damage (8–10).

Phytoplankton experience turbulence as microscale fluctuations in fluid velocity gradients, or “eddies” (8), which transport and randomly reorient cells every few seconds (the Kolmogorov timescale). When coupled with motility, turbulence can create patchiness in the distribution of phytoplankton at millimeter to centimeter scales (the Kolmogorov length scale) (11, 12), potentially impacting on population ecology by modulating cell encounter rates and signaling. To cope with turbulence, phytoplankton can regulate lipid content, release of infochemicals, or gene expression profiles (13). On behavioral timescales, phytoplankton are able to actively respond to the fluid mechanical cues associated with turbulence (14) by regulating buoyancy (15) or switching migratory direction—presumably to avoid turbulent patches—by rapidly modulating their cellular morphology (16). In particular, some dinoflagellates and raphidophytes, including strains of *Heterosigma akashiwo*, *Chattonella subsalsa*, and *Prorocentrum minimum*, can alter their direction of vertical migration when exposed to the periodic changes of orientation relative to gravity caused by turbulent eddies, leading to the emergence of a downward-migrating subpopulation among cells originally migrating upward (16).

Vertical migration is a hallmark of many phytoplankton species (17), giving them access to light by day and nutrients at depth by night (18). To migrate through the water column, motile species use gravitaxis (19), a form of directed motility mediated by a stabilizing torque that biases swimming in or against the direction of gravity. The physiological mechanisms mediating the nexus between turbulence and vertical migration are thus key to understanding how the hydrodynamic environment shapes phytoplankton dynamics in today’s oceans as well as in future altered turbulence regimes induced by climatic changes (20). Yet, a fundamental understanding of the impact of turbulence on phytoplankton migratory behavior, physiology, and fitness is lacking.

**Fig. 1.**
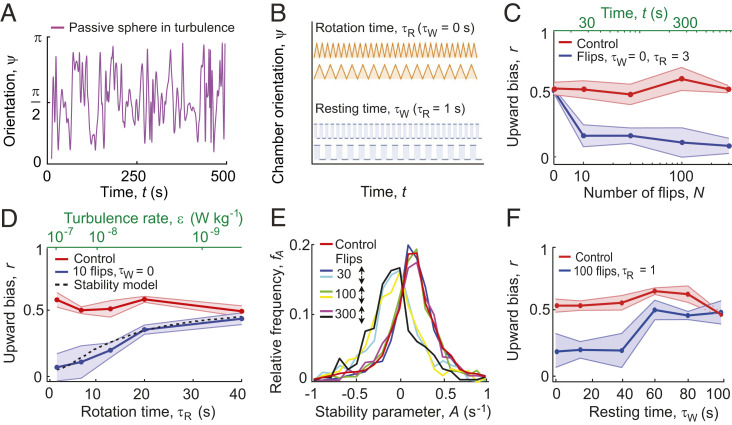
Rotation rate and resting time between reorientations relative to gravity determine the migratory response of *H. akashiwo* to turbulent cues. (*A*) Time series of the orientation, ψ(*t*), of a passive sphere relative to the vertical in a three-dimensional (3D) isotropic turbulent flow, obtained from a direct numerical simulation. The signal reveals the characteristic effects of the microscale turbulent eddies, that is, periods of time where the sphere abruptly changes its orientation by up to an angle π (modeled in this work by a rotation time), alternating with regions in which the orientation is more constant over time (modeled in this work by a resting time). (*B*) Experiments are based on a simplified characterization of intermittent turbulence in terms of two parameters: the rotation time, τ_R_, over which the experimental chamber completes a reorientation of amplitude π (one “flip” at a rate Ω = π/τ_R_), and the resting time, τ_W_, during which the chamber is held still between reorientations. Orange curves show the case with no intermittency (τ_W_ = 0 s) and blue curves show two cases of short and long resting times τ_W_. (*C*) The upward bias index, *r* (*Materials and Methods*), as a function of the number of flips, *N*, decreases from 0.52 to 0.17 over only 30 s of flipping (τ_R_ = 3 s, time elapsed *t = N τ_R_*). (*D*) The upward bias as a function of the rotation time, τ_R_, for a constant resting time, τ_W_ = 0 s (blue curve). Faster reorientations (smaller τ_R_), which correspond to stronger turbulence, ε, cause a larger population split when evaluated over the same number (10) of flips. Our model of cell stability (dashed line, *Materials and Methods*) correctly predicts the upward bias (i.e., the fraction of downward-migrating cells that emerge for each treatment). (*E*) Relative distribution of the cells’ mechanical stability, expressed as the stability parameter *A*. The red curve corresponds to a population of cells before flipping. Other colors correspond to cells from the top (↑) and bottom (↓) subpopulations after *n* = 30, 100, and 300 flips (τ_R_ = 3 s; τ_W_ = 15 s). (*F*) The upward bias as a function of the resting time, τ_W_, for a constant rotation time, τ_R_ = 1 s (blue curve). Shorter resting times (smaller τ_W_) induced a larger population split when evaluated over the same number (100) of flips. In *C*, *D*, and *F*, circles and shaded regions denote mean ± SD of four replicate experiments, and corresponding controls (measured over the same time period, but without flipping) are shown in red.

Here, using a combination of millifluidics-based visualization, quantification of stress accumulation, photophysiology, and mathematical modeling, we report that the emergent migratory behavior of the marine raphidophyte *H. akashiwo* exposed to turbulent cues is determined by the integration of reactive oxygen species (ROS) signaling. We used time-lapse imaging to track the migration of individual cells of *H. akashiwo* in a small (12 mm × 4 mm × 1.6 mm) rotating chamber (16) that can be rotated around a horizontal axis by a computer-controlled motor with any user-defined time series of the rotation angle. *H. akashiwo* is a ubiquitous coastal species (21) known for its allelopathic effects and toxic blooms (22) and frequently used as a model system in studies of vertical migration (23, 24). We performed experiments for different rotation time series, as a model system to determine the effect of the magnitude and intermittency of small-scale turbulent eddies (25). Ocean turbulence is often intermittent or patchy and its magnitude highly variable, with turbulent kinetic energy values ranging from ε = 10^−10^ to 10^−5^ W · kg^−1^ (5, 8), which correspond to Kolmogorov timescales τ_K_ = 100 to 0.3 s. Our experimental system models intermittent turbulence as a sequence of reorientations of the chamber of magnitude π, each taking a time τ_R_ separated in time by a resting time τ_W_ during which the chamber is kept still (Fig. 1 *A* and *B*). We hypothesized that the interplay between the rotation rate, Ω = π/τ_R_, and the time available for recovery, τ_W_, would regulate the emergence of the downward-migrating subpopulation from an initially upward-migrating population.

## Results

### Mechanical Stability in Phytoplankton Modulates Migratory Behavior in Response to Turbulent Cues.

The change in migratory behavior for a monoclonal population of *H. akashiwo* (CCMP452) occurred within the first 10 overturning events (Fig. 1*C*), corresponding to only tens of seconds at the highest rotation rate used (Ω *=* 1 rad · s^−1^, equivalent to a turbulence intensity ε = 10^−7^ W · kg^−1^; *Materials and Methods*). Exposure to additional reorientations had no further effect on migration, where the percentage of cells swimming upward changed from 77.5% before treatment (control) to 54.5% after 300 reorientations, with two almost equally abundant subpopulations: one continuing to swim upward and the other that switched to downward migration. The saturation of the behavioral response is also shown by the stable value of the upward bias index *r* over 10 (*r* = 0.17 ± 0.11) to 300 (*r* = 0.09 ± 0.06) reorientations (ANOVA, *F*_3,13_ = 0.26, *P* = 0.85). The upward bias index, *r* = (*f*_↑_ − *f*_↓_)/(*f*_↑_ + *f*_↓_), measures the relative proportions of upward-migrating (*f*_↑_) and downward-migrating (*f*_↓_) cells, and is quantified once flipping ceases and the population has had time to reach a stationary distribution inside the chamber (*Materials and Methods*). Varying the rotation rate Ω (0.08 rad · s^−1^ ≤ Ω ≤ 1 rad · s^−1^) for a fixed number of 10 reorientations with no resting time (τ_W_ = 0 s) changed the proportion of downward-migrating cells (Fig. 1*D*). At the fastest rotation rate tested (Ω *=* 1 rad · s^−1^), the highest concentration of downward-migrating cells was observed (*r* = 0.07 ± 0.10), while at the slowest rotation rate tested (Ω = 0.08 rad · s^−1^), the upward bias index (*r* = 0.44 ± 0.05) was not different from the nonrotating control experiment (*r* = 0.49 ± 0.05; *t*_8_ = 0.93, *P* = 0.38) (Fig. 1*D*). These results show that the stronger disturbances associated with faster reorientations triggered a stronger response and more cells actively changed their direction of migration relative to gravity.

The initial stability of a cell regulates how the cell is affected by reorientations. The mechanical stability of a cell, which is typically produced by an asymmetry in the cell shape or a nonuniform distribution of cell density (16, 19), allows the cell to maintain its orientation with respect to gravity. It can be measured by the stability parameter *A* = (2*B*)^−1^, where *B* is the characteristic time for the cell to rotate back to its vertical equilibrium orientation once perturbed from it. An analysis of the stability of a cell in an eddy with rotation rate Ω predicts that if |Ω| < |*A*|, the cell will swim with a constant angle relative to gravity (of angle θ_eq_ = arcsin(Ω*A*^−1^) for an upward-migrating cell or θ_eq_ = π – arcsin(Ω*A*^−1^) for a downward-migrating cell), whereas if |Ω| > |*A*|, the cell will tumble in a periodic orbit with period *T*_B_ = 2π (Ω^2^ – *A*^2^)^−1/2^ (26) (*Materials and Methods*). From this analysis, we can predict the fraction of cells that will tumble under the effect of reorientations and therefore switch their direction of migration from upward to downward as a function of the rotation rate Ω and the initial distribution of mechanical stabilities within a population (Fig. 1 *D* and *E*). We measured experimentally the initial distribution of mechanical stabilities for CCMP452 at the single-cell level (Fig. 1*E* and *Materials and Methods*), yielding a distribution of the stability parameter characterized by high variability (*A*_452_ = 0.09 ± 0.21 s^−1^). From the distribution of the cells’ initial stability parameter, our stability model correctly predicts the fraction of downward-migrating cells that emerge for each reorientation treatment (Fig. 1*D* and *SI Appendix*, Fig. S1). To further support our prediction that downward migration emerges when cells become destabilized by the fluid flow, we performed experiments on a second *H. akashiwo* strain (CCMP3374), which has higher mean stability than CCMP452 (*A*_3374_ = 0.23 s^−1^; *SI Appendix*, Fig. S1*A*). We observed that CCMP3374 cells shift to downward swimming at higher rotation rates compared to CCMP452 cells (*SI Appendix*, Fig. S1*B*) but likewise where |Ω| > |*A*| (*SI Appendix*, Figs. S1*C* and S2*A*). The cell’s mechanical stability thus imposes an amplitude filter on the local vorticity of the turbulence signal, whereby only reorientations with rotation rates Ω faster than the stability parameter *A* cause an upward-migrating cell to tumble (*SI Appendix*, Figs. S1*C* and S2*A*) and can thus trigger the emergence of downward migration.

### The Migratory Switch Is Mediated by a Bistability in the Stress Response.

The migratory behavior was further affected by the resting time τ_W_, a measure of the signal’s intermittency (Fig. 1*B*). This was revealed by experiments with fast reorientations (Ω = 3.14 rad · s^−1^), which induce a population split in CCMP452 in the absence of resting time (Fig. 1*D* and *Materials and Methods*). When the resting time was varied in the range τ_W_ = 0 s to 100 s, we found the population split to occur for values of τ_W_ below a threshold of 40 s (Fig. 1*F*), a value in line with the typical interval between reorientations experienced by CCMP452 cells in strong turbulence (*SI Appendix*, Fig. S2 *B* and *C*). A threshold response is characteristic of stress responses in eukaryotes (27), including dinoflagellates (28) and diatoms (29), and led us to hypothesize that a progressive intracellular accumulation of oxidative compounds resulting from the reorientations is the physiological mechanism underlying the change in migration direction.

**Fig. 2.**
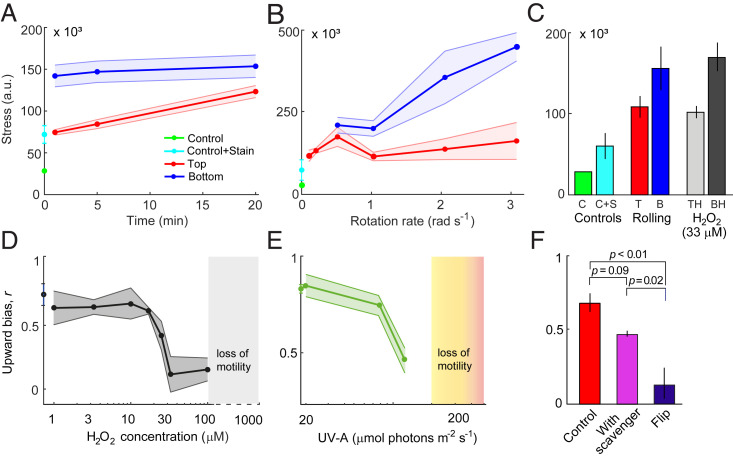
Bistability in oxidative stress mediates vertical migration of *H. akashiwo*. Oxidative stress level, caused by intracellular ROS accumulation, is shown as a function of (*A*) the time exposed to rolling (Ω = 1 rad · s^−1^) and (*B*) the rotation rate (rolling time *t* = 5 min). Curves show the increase in oxidative stress levels for the top (red, “Top”) and the bottom (blue, “Bottom”) subpopulations. Also shown are the baseline fluorescence signal of untreated control cells (green, “Control”) and of control cells treated with the fluorescent stain CM-H_2_DCFDA, a general oxidative stress indicator (cyan, “Control+Stain”). Stress levels were computed from flow cytometric measurements (mean ± SD of at least three replicates). (*C*) Oxidative stress level caused by intracellular ROS accumulation after exposure to exogenous H_2_O_2_ or to 20 min of rolling (Ω = 1 rad · s^−1^). Controls (same as in *A* and *B*) are shown in green and cyan. Bars show the oxidative stress levels for the top (red, “T” for rolling; light gray, “TH” for H_2_O_2_) and the bottom (blue, “B” for rolling; dark gray, “BH” for H_2_O_2_). Stress levels (a.u., arbitrary units) were computed from flow cytometric measurements (mean ± SD of three replicates). Cells that switch to downward-swimming behavior show elevated stress levels (“B” and “BH”), while cells that continue to swim upwards (“T” and “TH”) show stress levels closer to those of the controls. (*D*) Upward bias as a function of the concentration of H_2_O_2_ added to the medium 30 min before measurements. H_2_O_2_ concentrations above 15 μM elicit the population split in migration direction. Above 100 μM H_2_O_2_, cells lost motility (*SI Appendix*, Fig. S12*B*). (*E*) Upward bias as a function of the intensity of UV-A light (emission peak = 395 nm) applied for 30 min. Photon flux densities above 80 µmol photons m^−2^ · s^−1^ elicit the population split in migration direction. Above 200 µmol photons m^−2^ · s^−1^, cells lost motility (*SI Appendix*, Fig. S12*C*). (*F*) Upward bias for cells kept in still conditions (red bar, “Control”), for cells that were flipped (blue bar, 100 flips, Ω = 1 rad · s^−1^, τ_W_ = 0 s), and for cells that were flipped after having been cultured in the presence of a scavenger of ROS (100 μM KI, magenta). Populations differed significantly in upward bias (one-way ANOVA, *F*_2,7_ = 19.8, *P* = 0.001). Brackets show *P* values from post hoc Tukey’s honest significant difference (HSD) tests (*SI Appendix*, Table S1). For all panels, data shown correspond to mean ± SD of three replicates.

To test this hypothesis, we performed experiments with cells stained using a marker (CM-H_2_DCFDA) that forms a fluorescent compound in the presence of ROS, which are signaling molecules that mediate the perception of diverse environmental stress conditions (30). Intracellular ROS accumulation was quantified by flow cytometry (*SI Appendix*, Fig. S3 and *Materials and Methods*). For these experiments, we used continuous rotation on a roller device (τ_W_ *=* 0 s, *Materials and Methods*) with a sample volume (2 mL) larger than the millifluidic chamber (75 µL) and thus more suitable for analysis by flow cytometry. The migratory response was found to be independent of whether rotation was continuously in one direction (i.e., rolling) or alternating between clockwise and counterclockwise (i.e., flipping) (*SI Appendix*, Fig. S4). After just 1 min of rolling (Ω = 1 rad · s^−1^), downward-migrating cells were found to have accumulated twofold more ROS compared to upward-migrating cells, and the difference in the accumulated stress between the two subpopulations was consistently detected also after 5 min and 20 min of rolling (two-sample *t* test, *P* = 0.04; *t*_6_ = 2.5) (Fig. 2*A*). A similar stress response was detected in the other strain of *H. akashiwo*, CCMP3374, when exposed to the same treatment (*SI Appendix*, Fig. S5).

Additionally, we characterized the differences in the ROS accumulation between upward- and downward-migrating subpopulations as a function of the rotation rate of the rolling device. We found that for rotation rates of Ω < 1 rad · s^−1^, faster rotations caused a progressively higher ROS accumulation in the cells harvested at the top (Fig. 2*B*). For the range of rotation rates Ω < 1 rad · s^−1^, the subpopulation of upward-swimming cells contains both cells with high mechanical stability (*A* > Ω) and part of the cells with low mechanical stability (*A* < Ω) that accumulated ROS below the stress threshold for the switch in migratory direction. For rotation rates such that Ω ≥ 1 rad · s^−1^, the cells with low mechanical stability fully undertake the behavioral switch and accumulate progressively higher stress compared to upward-migrating cells (Fig. 2*B*). Taken together, these observations indicate that a bistability in oxidative stress response is associated with the split in migratory behavior of phytoplankton cells experiencing turbulent cues.

To further support the finding that ROS affects migration behavior, we observed the migration of cells exposed to different exogenous stressors known to cause ROS accumulation. In a first set of experiments, we added hydrogen peroxide (H_2_O_2_) to the medium. H_2_O_2_ diffuses across the cell membrane, mimicking the physiological intracellular accumulation of ROS caused by the reorientations. We determined the ROS levels induced by H_2_O_2_ for both *H. akashiwo* strains, and we compared those levels with the ROS levels generated by exposure to 20 min of rolling. The results show that the stress levels generated in the upward- and downward-migrating subpopulations after exposure to H_2_O_2_ (at a concentration *C* = 33 µM) quantitatively match the ROS accumulation observed upon rolling, where we observed a bistable stress response (Fig. 2*C*). Exposure to exogenous H_2_O_2_ induced the population split in migratory behavior above a threshold concentration of 15 µM H_2_O_2_ (Fig. 2*D*), with a threshold-like behavioral response akin to that caused by fast reorientations (Fig. 1*F*). Most notably, the ROS levels of CCMP452 cells increased sharply upon increasing the concentration of exogenous H_2_O_2_ from 10 to 33 µM (*SI Appendix*, Fig. S6), a concentration range that coincides with the H_2_O_2_ concentration causing the population split (Fig. 2 *C* and *D*). In a second set of experiments, we exposed CCMP452 cells to light for 30 min at intensities known to lead to ROS accumulation (31) and characteristic of ocean surface waters (32). We observed the emergence of a downward-migrating subpopulation for cells exposed to near-UV-A light (380 to 400 nm) at intensities greater than 80 µmol photons m^−2^ · s^−1^ or to full-spectrum light (320 to 800 nm) at intensities greater than 650 µmol photons m^−2^ · s^−1^ (Fig. 2*E* and *SI Appendix*, Fig. S7*A*). Finally, we repeated the overturning experiments for cells pretreated with the ROS scavenger potassium iodide (KI; *Materials and Methods*) at an exogenous concentration of 100 µM (Fig. 2*F* and *SI Appendix*, Table S1). No emergence of a downward-migrating subpopulation was observed in this case. To summarize, this suite of experiments, in which we exposed cells to ROS scavengers and inducers, showed that the behavioral response could be blocked by adding a ROS scavenger (KI) to the medium and that the behavioral response could be triggered by applying external ROS (in the form of H_2_O_2_ and high irradiance) that activated the response downstream in the signaling cascade. Taken together, these experiments demonstrate the causal link between intracellular stress accumulation mediated by ROS and the behavioral switch in migration direction.

To determine the dependence of the stress threshold above which downward-swimming emerges on cell physiology, we conducted rolling experiments with cell cultures under different conditions. Because ROS production patterns in raphidophytes vary across different growth phases (33–35), we investigated the dependence of the stress threshold by performing rolling experiments using a population at 72 h after inoculation in the fresh medium (early exponential phase) to compare to the standard treatment between 96 h and 120 h after inoculation (midexponential phase) (16). While the stress accumulation in *H. akashiwo* after exposure to rolling is regulated by the growth phase, we found that the value of the stress threshold relative to the baseline stress level is conserved across different growth phases (*SI Appendix*, Fig. S8). Before rolling, cells in early exponential phase presented a higher baseline ROS production rate compared to cells in mid-exponential phase. After rolling, the accumulated stress and the value of the threshold were higher in early exponential phase (*SI Appendix*, Fig. S8*A*). These results are the consequence of a less-efficient scavenging machinery and an increased endogenous ROS production, in line with the literature on ROS production patterns in raphidophytes (33–35). However, the increase in the ROS after rolling, *s*, relative to the baseline stress level, *s*_0_, is comparable between the two growth phases for both upward- and downward-migrating subpopulations (*SI Appendix*, Fig. S8 *A*, *Inset*). This analysis allowed us to identify the threshold *h* = *s*/*s*_0_ = 2.3 ± 0.6 for the emergence of the migratory switch, because below this value of relative stress, cells still perform upward swimming after exposure to rolling.

### Phytoplankton Navigation under Turbulence Is Regulated by Stress Accumulation–Dissipation Dynamics.

**Fig. 3.**
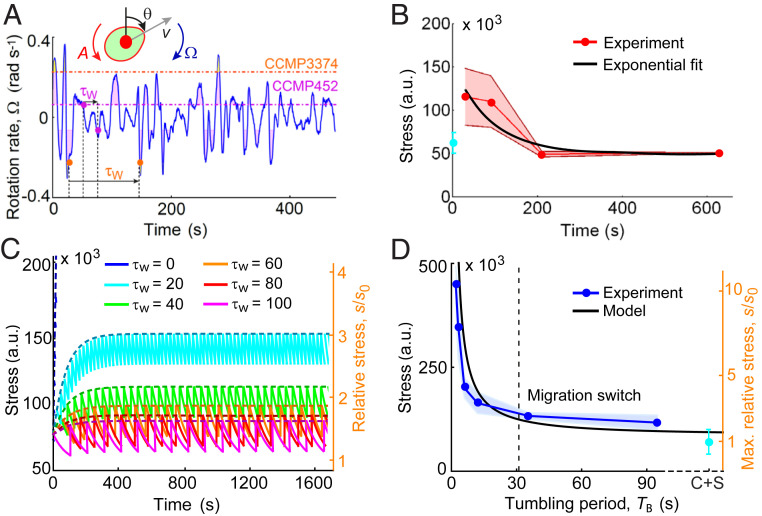
The dynamics of ROS accumulation–dissipation regulate migratory behavior of *H. akashiwo*. (*A*) Time series of the rotation rate relative to gravity, Ω, of a passive cell in a 3D isotropic turbulent flow obtained from a direct numerical simulation. Dashed lines represent the value of the stability parameter, *A*, for CCMP452 (magenta) and CCMP3374 (orange). Shaded regions represent time windows over which a cell will be tumbled by turbulence (i.e., |Ω| > |*A|*, *SI Appendix*, Fig. S2*A*), corresponding in our experiments to imposed reorientations. During times when the rotation rate is |Ω| < |*A*|, cells are not tumbled but will achieve an equilibrium swimming orientation (*SI Appendix*, Fig. S1*C*), corresponding in our experiments to a resting time, τ_W_, between reorientations. The higher mechanical stability of CCMP3374 results in longer resting times (*SI Appendix*, Fig. S2 *B* and *C*). (*B*) The stress dissipation dynamics, measured in still conditions (red, mean ± SD of four replicates) for a population previously exposed to 5 min of rolling (Ω = 1 rad · s^−1^), is characterized by an exponential decay with timescale τ_S_ = 87 ± 32 s (black). (*C*) Time series of stress (Eqs. **3** and **4**, *Materials and Methods*) predicted by the mathematical model for the same range of resting times investigated experimentally (Fig. 1*F*). Cells rapidly accumulate stress after being reoriented (Ω = 3.14 rad · s^−1^) and dissipate it with timescale τ_S_. The dashed curves represent the upper envelope of the stress signal (*SI Appendix*, Eq. **S6**). Stress values have been also normalized by the baseline stress level, *s*_0_ = 42 × 10^3^ a.u., for a population under still conditions (orange *y* axis). (*D*) Stress caused by intracellular ROS accumulation in a population exposed to rolling as a function of the tumbling period, which is set by the rotation rate through the expression *T*_B_ = 2π (Ω^2^ – *A*^2^)^−1/2^, with *A* = 0.09 rad · s^−1^ the stability parameter of CMMP452. Stress data are taken from Fig. 2*B* for the subpopulation of cells that undertake the switch in the direction of migration. The black line shows the predicted maximum stress after rolling (*SI Appendix*, Eq. **S5**). The vertical dashed line corresponds to the tumbling period *T*_B_ = 32 ± 13 s at which the threshold value for the migration switch (*SI Appendix*, Fig. S8*A*), *h* = 2.3 ± 0.6 (relative stress *s*/*s*_0_, orange *y* axis), is reached. The value of the stress spike generated at each tumbling event is a fitting parameter of the model, Δ*s/s*_0_ = 0.40 ± 0.07. The cyan dot (C + S) represents the control in which cells were treated with the fluorescent stain CM-H_2_DCFDA.

To predict the emergence of the behavioral switch in the migratory response, we devised a mathematical model of stress dynamics in cells exposed to turbulence. In the model, the cell’s mechanical stability prevents overturning by weaker eddies (Fig. 1*D* and *SI Appendix*, Figs. S1*C* and S2*A*), thus creating resting times between periods during which the cell is overturned (Fig. 3*A* and *SI Appendix*, Fig. S2 *B* and *C*). Accordingly, a cell accumulates ROS whenever it is tumbled by an eddy and dissipates stress by means of its intracellular antioxidant capacity (34, 36), with a characteristic dissipation timescale τ_S_ (*Materials and Methods* and Eqs. **3** and **4** and *SI Appendix*). During a tumbling event, a cell is rapidly reoriented relatively to gravity, and it experiences an impulsive force of typical magnitude *F*_g_ ∼ 1 pN (*SI Appendix*). We assumed that, when tumbling, intracellular stress is generated in the cell in the form of a nearly instantaneous release (i.e., a spike) of ROS of amplitude Δ*s* (a free parameter of our model, *Materials and Methods*). We quantified stress dissipation dynamics and the timescale τ_S_ experimentally by observing the reduction of ROS over time for cells after exposure to continuous rolling for 5 min (Ω = 1 rad · s^−1^). These experiments showed that stress decays exponentially over time with a timescale τ_S_ = 87 ± 32 s (Fig. 3*B*).

Using this model of stress accumulation–dissipation dynamics, we can predict the time series of stress accumulation for individual cells exposed to flipping (Ω = 3.14 rad · s^−1^) for the same range of resting times τ_W_ (Fig. 1*F*) or to rolling for the same range of rotation rates Ω (Fig. 2*B*) studied experimentally. This allowed us to compute the stress accumulated by cells during reorientations as a function of the resting time (Fig. 3*C*) or the tumbling period *T*_B_ (Fig. 3*D*) and to compare the model predictions with the experimentally measured ROS concentrations at which downward migration emerged (Fig. 2 *A* and *B*). We used the ROS accumulation as a function of the rotation rate to constrain the value of the parameter Δ*s*/*s*_0_ = 0.40 ± 0.07 (Fig. 3*D*), where the baseline level of stress *s*_0_ is quantified by observing the ROS production in the cells before rolling. By using the stress threshold *h* = 2.3 (obtained through rolling experiments; *SI Appendix*, Fig. S8 *A*, *Inset*) above which a cell would switch its migratory strategy, we find that the theoretical prediction for the maximum resting time in the flipping experiments (or equivalently for the maximum tumbling period in the rolling experiments; see *SI Appendix*) associated with the emergence of downward-migrating cells (τ_W_ = 32 ± 13 s, Fig. 3*D*) quantitatively matches the value of τ_W_ observed experimentally (τ_W_ = 40 s, Fig. 1*F*).

In order to model stress accumulation while capturing the effect of intrinsic variability in the mechanical stability and in the ROS scavenging efficiency, we developed an analytical model of stress accumulation during navigation under fluid rotations that accounts for heterogeneity in these two phenotypic traits (*SI Appendix*, Fig. S9 and *SI Appendix*). For the subpopulation performing upward swimming even after exposure to strong turbulence or high concentrations of H_2_O_2_, we implemented a smaller dissipation timescale. This follows from the observation of a bistable stress response upon induction with H_2_O_2_ without turbulence, in which the stability parameter does not play any role (Fig. 2*C*) (*SI Appendix*). Using the same fitting parameter Δ*s/s*_0_ = 0.40 estimated through the accumulated stress at the population scale (Fig. 3*D*) and the experimental distribution of the stability parameter (Fig. 1*E*), we found good agreement between the stress distributions for the two subpopulations in the experiments and in the stochastic model capturing single-cell variability (*SI Appendix*, Fig. S10).

Our model further predicts the temporal dynamics for the saturation of the stress response in which 98% of the total stress is accumulated within 5 min of rolling (at a rotation rate Ω = 1 rad · s^−1^), similarly to the stress saturation dynamics observed in the experiments (Fig. 2*A*). The timescale for saturation of the stress response is insensitive to changes in Δ*s*, which is a multiplicative factor in the model. Conversely, changing the ratio τ_W_/τ_S_ would change the number of rotations *N*, and therefore the time, needed to reach saturation of the stress response (*SI Appendix*, Eq. **S6**). The model reveals a criterion for the emergence of downward migration: when τ_W_/τ_S_ < 1, the stress increases hyperbolically as a function of the ratio τ_W_/τ_S_ (*SI Appendix*, Eq. **S8**), because the ROS scavenging machinery of a cell is too slow in dissipating stress relative to the rate at which stress accumulates owing to the short interval between reorientations by turbulence, and the accumulated stress induces the switch in migratory behavior.

### Brief Exposure to Turbulence Has Long-Lasting Effects on Phytoplankton Fitness.

**Fig. 4.**
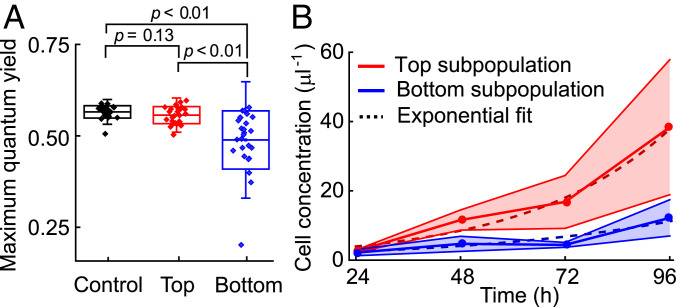
The oxidative stress induced by turbulent cues reduces photosynthetic efficiency and growth rate of *H. akashiwo*. (*A*) Maximum photosynthetic quantum yield, *F*_*v*_/*F*_*m*_*,* of *H. akashiwo* cells before and after turbulence-induced population splits. A PAM chlorophyll fluorometer was used to assess the maximum quantum yield of photosystem II on single cells collected from the top (*n* = 26) and bottom (*n* = 25) of the chamber after exposure to rolling (Ω *=* 1 rad · s^−1^) for 5 min and from cells not exposed to rolling (Control, *n* = 25). All cells were dark-adapted for 15 min before measurements. Boxes show ±1 SD, whiskers ±2 SD, and the central line indicates the mean. Control and flipped populations differed significantly (one-way ANOVA, *F*_2,74_ = 18.8, *P* < 0.001). Brackets show *P* values from post hoc Tukey’s HSD tests (*SI Appendix*, Table S2). (*B*) Increase in cell concentration with time for the subpopulations extracted from the top (red) and bottom (blue) of the chamber after exposure to rolling (Ω *=* 1 rad · s^−1^) for 5 min (mean ± SD of three replicates). Cells were regrown from the same initial density. The intrinsic growth rate, *g*, was quantified for each subpopulation by fitting an exponential function (dashed curves; *g*_↑_ = 0.74 ± 0.02 d^−1^, *g*_↓_ = 0.47 ± 0.03 d^−1^).

The accumulation of ROS in response to turbulent cues directly affected cell physiology for multiple cell divisions after cessation of the cue. Single-cell photophysiological measurements using pulse-amplitude–modulated chlorophyll fluorometry (PAM; see *Materials and Methods*) showed that the downward-migrating cells emerging after 5 min of continuous rolling (Ω = 1 rad · s^−1^) had 15% lower photosystem (PS) II photosynthetic quantum yields (*F*_*v*_/*F*_*m*_) compared to upward-migrating cells (Fig. 4*A* and *SI Appendix*, Table S2). This reduction may stem directly from endogenous ROS, which can reduce photosynthetic quantum yields (37) via the general suppression of PSII D1 protein synthesis and repair (38, 39), activation of nonphotochemical pathways, or photoinactivation of PSII reaction centers (40). This reduction in photosynthetic performance in *H. akashiwo* is acute when compared to the reductions of typically less than 10% caused by high light exposure in diatoms (31, 37). We further found evidence for longer-term physiological damage induced by 5 min of continuous rolling corresponding to strong turbulence (Ω = 1 rad · s^−1^), with the downward-migrating subpopulation exhibiting a 35% lower growth rate over 4 d (*g*_↓_ = 0.47 ± 0.03 d^−1^) than the upward-migrating subpopulation (*g*_↑_ = 0.74 ± 0.02 d^−1^) (Fig. 4*B*), with the growth of the latter comparable to the growth rate obtained for control cells (*g* = 0.69 ± 0.06 d^−1^) (*SI Appendix*, Fig. S11). We also estimated the instantaneous growth rate for the two subpopulations between 72 h and 96 h using log(*n*_96_/*n*_72_), where *n*_72_ and *n*_96_ are the cell concentrations at 72 h and 96 h after exposure to rolling. Within the time period 72 h to 96 h after exposure to turbulent cues, we observed a comparable growth rate in the two subpopulations (*g*_↑_ = 0.83 ± 0.39 d^−1^ for the upward-migrating subpopulation and *g*_↓_ = 1.01 ± 0.21 d^−1^ for the downward-migrating subpopulation). The growth reduction over multiple generations (approximately three) indicates that the reorientation-induced ROS accumulation has systemic consequences for *H. akashiwo* and suggests the potential presence of a transgenerational stress memory, akin to epigenetic effects observed in plants (41). This result is in contrast with stress propagation in *Escherichia coli* and yeast (27), where mother cells retain the oxidized aggregated protein, leaving daughter cells cleared of damaged proteins (36).

## Discussion

**Fig. 5.**
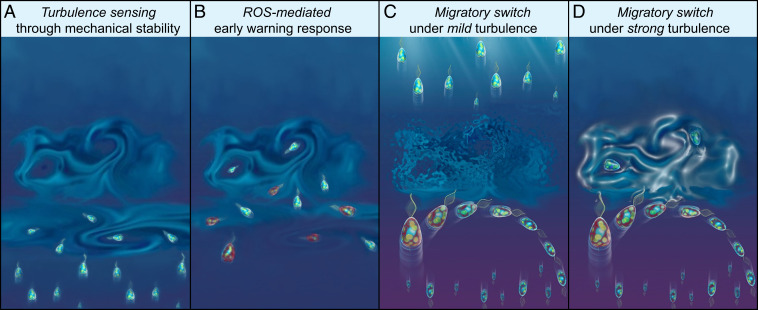
A ROS-mediated early-warning system could help *H. akashiwo* evade the damaging effects of turbulence. (*A*) A population migrating vertically by gravitaxis encounters a turbulent patch that disrupts the cells’ migratory pattern and could result in damage or death. Cells sense the intensity of a microscale turbulent eddy through their mechanical stability. Less stable cells are more easily thrown off balance and tumble under the effect of turbulence. (*B*) Cells integrate ROS signaling generated by tumbling events through a mechanism regulated by their scavenging machinery. Within timescales as short as tens of seconds, a bistable stress response mediates the emergence of two subpopulations with opposite migratory direction. Cells shown in red have accumulated higher levels of ROS. (*C* and *D*) The population splits into two subpopulations: one that continues to migrate upward and one that switches to downward migration to avoid the turbulence. Cells change the direction of their vertical migration by rapidly changing their cellular morphology, which, together with the internal distribution of organelles, governs the migration direction (16). (*C*) If the turbulence is weak (ε < 10^−6^ W · kg^−1^), cells from both subpopulations survive, with upward migrating cells continuing their journey to well-lit surface waters to photosynthesize and divide. (*D*) If the turbulence is strong (ε > 10^−6^ W · kg^−1^), upward-swimming cells may get trapped, lose motility, and possibly perish. By avoiding the turbulent patch, the downward-swimming cells suffer a reduction in growth because of elevated levels of ROS and a reduction of photosynthetic efficiency, but provide a reservoir to re-establish the population. In this manner, the behavioral split into two subpopulations creates an evolutionary advantage for the population overall.

The evidence we have presented for the role of stress in vertical migration provides a view of the ecological implications of the active response of phytoplankton to turbulence. Our results demonstrate that the overturning of cells, a fundamental yet to-date-unappreciated mechanical cue due to turbulence in the ocean, can trigger behavioral and physiological responses over timescales spanning tens of seconds to multiple generations. The good agreement between our model and observations suggests that motile phytoplankton use mechanical stability to sense the intensity of turbulent cues (Fig. 5*A*) and integrate these cues in time via ROS signaling (Fig. 5*B*): when ROS accumulates beyond a threshold, it triggers the switch in migratory behavior (Fig. 5 *C* and *D*) underpinned by a rapid modulation of the cellular morphology, which, together with the internal distribution of organelles, determines the sign of the stability parameter and thus the direction of migration (16). This ROS-mediated early-warning strategy may be advantageous owing to the heterogeneity in mechanical stability within monoclonal populations (Fig. 1*E*). The reorientations used in our experiments, corresponding to moderate to strong levels of turbulence (ε = 10^−9^–10^−6^ W · kg^−1^, *Materials and Methods*), did not inhibit motility (*SI Appendix*, Fig. S12*A*). By responding to the ROS-mediated early warning upon first encountering a region of turbulence, cells with weaker mechanical stability will avoid swimming into the “eye of the storm” where they could get trapped (42), damaged, or lose motility (8–10) (Fig. 5*D*). Heterogeneity is also seen in the antioxidant capacity, potentially the product of a tradeoff in which cells with low antioxidant capacity are more sensitive to turbulent cues via ROS signaling but at the cost of weaker protection against other environmental cues eliciting oxidative stress. The cells belonging to the upward-migrating subpopulation might have a potentiated scavenging machinery (represented in our model by a shorter timescale τ_S_) and/or a decreased baseline stress rate. The increased scavenging machinery in part of the population could originate as natural variability (43) or could be linked to a different phase of the cell cycle, which in a population in exponential phase is dictated by the time from the last division.

The emergence of downward migration upon exposure to well-known ROS inducers (H_2_O_2_, UV-A radiation, and high irradiance), in the absence of turbulent cues, shows that ROS accumulation is the cause of the migratory response but at the same time begs the question of how specificity of ROS signaling (30, 36) toward turbulence might be achieved in *H. akashiwo*. In fact, specificity of response may not be necessary if avoidance is a universally appropriate response to accumulation of ROS. Elevated levels of exogenous H_2_O_2_ (100 µM), UV-A radiation (300 µmol photons m^−2^ · s^−1^), and full-spectrum light (650 µmol photons m^−2^ · s^−1^) negatively impacted motility (*SI Appendix*, Figs. S7*B* and S12 *B* and *C*). Exposure to excessively high levels of irradiance can cause photoinhibition (31, 37), and downward migration would be a relevant response in the upper layers of the ocean where cells can experience light exposure similar to the levels used in our experiments (32).

Despite the short timescales we have observed in the behavioral and physiological response of cells to turbulence (tens of seconds to minutes), the physiological ramifications and acclimation of plankton to environmental signals in the ocean can be long term (4). Our finding that there is a growth disadvantage for cells that switch migratory behavior upon flipping (Fig. 4*B*) would result in a considerable difference in cell number after only a few divisions. By considering two subpopulations with the same initial number of cells and with growth rates *g*_↑_ = 0.74 d^−1^ and *g*_↓_ = 0.47 d^−1^ for upward and downward migration, the downward-migrating subpopulation would grow by only 34% as many new cells compared to the upward-migrating subpopulation after *t* = 4 d, calculated as exp(*g*_↓_*t*)/exp(*g*_↑_*t*). A reduction in the growth rate of the emergent downward-migrating subpopulation indicates that even brief exposure to strong turbulence, in the order of hundreds of seconds, can induce lasting physiological changes similar to those induced by longer exposure to other environmental factors such as intense light (31, 32, 36, 37), temperature (44), or nutrient availability (2, 4, 33, 45). Finally, our results suggest that global warming will not only impact phytoplankton physiology and metabolism directly but also indirectly through their response to decreased mean turbulence intensities and more energetic local storm events (20, 45). We propose that these changes in the physical regime of the water column will favor adaptive strategies that allow cells to cope with the stochasticity of turbulence (46). One example of this is the response described here for a harmful-algal-bloom–forming species, mediated by high phenotypic variability in swimming mechanics and by ROS bistability. Deepening our understanding of the physiological mechanisms underpinning these adaptive strategies, exemplified in our work through the mechanism of a ROS-mediated warning system, will thus contribute to the understanding of the responses of migrating populations and ultimately of community composition in future ocean conditions.

## Materials and Methods

### Cell Culture and Growth Rate Measurements.

Two strains of the raphidophyte *H. akashiwo* (47) were examined: CCMP452 and CCMP3374 (both obtained from the National Center for Marine Algae and Microbiota, Bigelow Laboratory for Ocean Sciences, Maine). Cells were cultured in 50-mL sterile glass tubes under a diel light cycle (14 h light: 10 h dark; 75 μmol photons m^−2^ · s^−1^) in f/2 (minus silica) medium, at 21 °C (CCMP452) or 18 °C (CCMP3374). For propagation of the cell cultures, 2 mL of the parent culture was inoculated into 25 mL of fresh medium every 2 wk. Experiments with CCMP452 were carried out with monoclonal cell cultures that were grown from a single parent cell isolated using an inoculation loop (diameter of ∼100 μm) developed in-house. The loop was dipped into a culture to trap a thin liquid layer, and microscopy was used to select the cases with only a single CCMP452 in the layer. Each individual cell was transferred to a separate well in a 36-well plate containing fresh growth medium. Experiments were conducted between 96 h and 120 h after inoculation, which corresponds to the early exponential growth phase of CCMP452 (16) at room temperature (21 °C). A fixed period of the day (between the hours of 09:00 and 15:00) was chosen for the experiments to rule out any possible artifacts due to the diurnal migration pattern of many phytoplankton species, including *H. akashiwo* (16, 48). For the experiments to test the effect of scavengers on the oxidative stress–induced split, CCMP452 cells were grown with KI, an H_2_O_2_ scavenger (49, 50). Cells in preliminary trials were grown in suspensions containing a range of KI concentrations: 1 μM, 10 μM, 100 μM, and 1 mM. All scavenging experiments reported here were performed with the 100-μM KI concentration. At this concentration, the growth rate, vertical distribution, and swimming speed of CCMP452 cells matched those of the control cell culture (cells grown without KI) in the absence of turbulence treatments.

To study the long-term impact of turbulent cues on growth rates, both the top and bottom subpopulations of CCMP452 were harvested (300 μL each) from a 2-mL cell culture vial that had been exposed to turbulent cues in the form of rolling for 5 min (*SI Appendix*, *Generation of turbulent cues, (*ii*) Rolling*). Prior to harvesting the subpopulations, the cell culture was allowed to attain the postturbulence stationary vertical distribution driven by their migration behavior (*SI Appendix*, *Upward bias index*). The harvested cells were introduced into the supernatant of the initial cell culture (from which the 2-mL suspension had been taken) in a 1:12 ratio and allowed to grow over 96 h (Fig. 4*B*). Cells were counted every 24 h by flow cytometry (CytoFLEX S, Beckman Coulter), and in parallel, their motility was checked using phase-contrast microscopy (Nikon Ti-E, Nikon, Japan). The cell concentration of each of the subpopulations was fitted over the 96-h period using the least squares method to obtain exponential growth curves (Wolfram Mathematica version 11.3, Champaign, IL).

### Quantification of Endogenous Stress Production.

To quantify the accumulation of ROS, cells exposed to turbulence or static conditions were incubated under dark conditions for 30 min in 10 μM CM-H_2_DCFDA (Ex/Em: ∼492 to 495/517 to 527 nm, Thermo Fisher Scientific, diluted in f/2). CM-H_2_DCFDA is a chloromethyl derivative of H_2_DCFDA that enables the detection of low concentrations of ROS. The marker is a suitable indicator for long-term quantification of ROS as it passively diffuses into live cells and forms a highly stable fluorescent adduct when oxidized. After the 30-min incubation period, fluorescence intensities of single cells were quantified using a flow cytometer (CytoFLEX S, Beckman Coulter), in the FITC-A channel (Ex/Em: ∼488/520 nm). Single-cell oxidative stress levels are represented as (relative) fluorescence units. To obtain the stress levels, we subtracted the FITC-A values for the control in the absence of CM-H_2_DCFDA staining from the FITC-A values for the stained cells. This additional step ensured the subtraction from the stress measurement of the autofluorescence of *H. akashiwo* over the green portion of the spectrum, characteristic of raphidophytes (51). Fluorescence levels were obtained for the turbulence-exposed population for the top and bottom subpopulations and for the control population (no turbulence) with and without the addition of CM-H_2_DCFDA.

### PAM Chlorophyll Fluorometry Experiments.

PAM was used to quantify the photosynthetic performance of cells after exposure to turbulent cues. Microscopic multicolor-variable chlorophyll fluorescence imaging (IMAG-RGB; Heinz Walz GmbH, Effeltrich, Germany) was used to quantify the photosynthetic activity of individual cells of CCMP452. A detailed technical description of the microscope system can be found elsewhere (52). For PAM measurements, cells were placed into one of the channels of a prefabricated glass-bottom microfluidic chamber with a depth of 100 µm (Ibidi µ-Slide VI, Ibidi GmbH, Martinsried, Germany). Using the saturation pulse method (40, 53), which is based on recording fluorescence yields before and during a saturating light pulse, the maximum quantum yield of photosynthetic energy conversion in PS II, *F*_*v*_/*F*_*m*_ = (*F*_*m*_ – *F*_*0*_)/*F*_*m*_, was measured after a 15-min dark incubation. Photosynthetically active radiation (PAR, 400 to 700 nm) was provided by coalescing red, green and blue (RGB) light-emitting diodes (LEDs), which were calibrated before each experiment using a PAR light-sensor (MC-MQS micro quantum sensor, Walz, Effeltrich, Germany) connected to a universal light meter (ULM-500, Walz). All measurements were performed using coalesced RGB LEDs (“white light”).

### Gravitactic Cells in a Fluid under Solid Body Rotation.

In Stokes flow regime (Reynolds numbers <1), the direction in which a gravitactic cell swims is at any instant determined by the balance of viscous and gravitational torques on the cell. For gravitactic cells characterized by a stability parameter *A* swimming at low Reynolds numbers in a fluid under solid body rotation in the vertical plane at a constant rotation rate Ω *=* π/τ_R_ (rad · s^−1^), which here exemplifies the characteristic reorientation rate 1/τ_K_ by Kolmogorov-scale turbulent eddies in the ocean, the equation of motion in the laboratory frame of reference reads

dθ/dt=–Asinθ+Ω, [1]

where θ measures the cell orientation to the vertical (*SI Appendix*, Figs. S1*C* and S2*A*). We have used the relation between the vorticity and the rotation rate for a fluid in a continuous (clockwise) solid body rotation in the vertical plane performed by the flipping chamber (and the rolling device), with the strain rate set to *E* = 0 (26). A gravitactic cell may therefore swim at a nonzero angle θ_eq_ = arcsin(Ω*A*^−1^) for upward-migrating cells (and θ_eq_ = π – arcsin(Ω*A*^−1^) for downward-migrating cells) relative to the vertical if |Ω| < |*A*| with 0 < θ_eq_ < π/2, or it may tumble if Ω is sufficiently large (|Ω| > |*A*|) and thus perform a periodic orbit with period

$$T_{\text{B}}=2\pi {\left(\Omega^{2}–A^{2}\right)}^{-1/2},$$ [2]

where *B =* (2*A*)^−1^ is the stability timescale (19). The solutions for Eq. **1** are portrayed in *SI Appendix*, Fig. S1*C* (Ω *=* 0.2 rad · s^−1^) for two different stability parameters corresponding to the two strains CCMP452 (low stability, where cells tumble) and CCMP3374 (high stability, where cells swim at an equilibrium angle θ_eq_). By applying the condition for tumbling |Ω| > |*A*| for the distribution of the stability parameter *f*_*A*_, measured experimentally (Fig. 1*E*), the fraction of cells migrating upward *f*_↑_ and downward *f*_↓_ can be extracted (*SI Appendix*), where we assumed, based on the saturation of the behavioral response after 10 flips only (Fig. 1*C*), that all tumbling cells undertake the switch in the migratory direction. We can then derive the upward bias *r* as a function of the rotation time τ_R_ of the chamber. The result of this stability analysis is plotted in Fig. 1*D* (black dashed line).

### Stress Dynamics of Gravitactic Cells under Turbulent Cues.

Above some critical value of rotation rate Ω_c_ at which |Ω_c_|> |*A*|, the cell tumbles by fluid shear in the vertical plane and performs periodic orbits because of a low mechanical stability (*SI Appendix*, Fig. S1*B*). An effect of rotation is that the gravitational acceleration g appears to be rotating in the rotating frame of reference. During a tumbling event, we assumed that intracellular stress is generated in the cell in the form of a nearly instantaneous release (i.e., a spike) of ROS whenever the cell is being reoriented relative to gravity (*SI Appendix*). In our model, the ROS spike specifically occurs at times *t*_*i*_ whenever the cell swims in a direction θ = π/2, that is, in the direction perpendicular to the gravity vector. This particular choice of swimming direction is arbitrary, and we could choose any value between π/2 < θ < π without changing our results. The intracellular scavenging machinery of the cells dissipates the accumulated stress *s* with a characteristic timescale τ_S_ (measured experimentally, Fig. 3*B* and *SI Appendix*). The resulting intracellular stress accumulation–dissipation dynamics are captured by the following differential equation

$$ds/dt=\sum_{t_{i}}\Delta s_{i}\text{δ}\left(t-t_{i}\right)–s\tau_{\text{S}}^{-1}+c_{0},$$ [3]

where the Dirac delta function δ(*t − t*_*i*_) records the stress spikes Δ*s*_*i*_ (assumed to all have the same value Δ*s*) occurring at times *t*_*i*_ for a given swimming trajectory and *c*_0_ is the baseline stress rate. Eq. **3** can be solved by performing the Laplace transform, which gives the stress level as a function of time

$$s\left(t\right)=\sum_{t_{i}}\Delta s\text{θ}\left(t-t_{i}\right){\text{e}}^{-\left(t-t_{i}\right)\tau_{\text{S}}^{-1}}+s_{0},$$ [4]

where θ(*t − t*_*i*_) is the Heaviside function and *s*_0_ = *c*_0_ τ_S_ is the baseline stress level before the fluid rotation. The population stress levels were measured with a flow cytometer for controls (no rotations) and after rolling (Fig. 2 *A* and *B*). By taking the partial sum in the summation in Eq. **4**, the upper envelope of the stress signal over time experienced by the tumbling cells after *N* periodic reorientations is

$$s_{\text{max}}\left(t\right)=\Delta s\frac{\left(1–{\text{e}}^{-NT_{\text{B}}\tau_{\text{S}}^{-1}}\right)}{\left(1–{\text{e}}^{-T_{\text{B}}\tau_{\text{S}}^{-1}}\right)}+s_{0},$$ [5]

where the sequence of times *t*_*i*_ at which stress is generated is *S* = {*T*_B_*,…iT*_B_*,…NT*_B_}. *T*_B_ is the period of the orbit for the tumbling cells given in Eq. **2**, which depends on the stability parameter *A* and on the rotation rate Ω. In *SI Appendix*, *Supplementary Text*, we further model the stress dynamics for cells exposed to turbulent cues for the two paradigmatic cases that we employed experimentally: 1) a continuous solid body rotation (i.e., rolling) with rotation rate Ω = π/τ_R_ (no resting phases, τ_W_ = 0), and 2) multiple, fast reorientations of amplitude π (i.e., flipping) occurring at a rate Ω >> *A*, alternating with resting phases captured by the timescale τ_W_ (Fig. 1*B*). To take into account population heterogeneity in the mechanical stability and stress dissipation parameters observed experimentally, we also derived a stochastic model of stress dynamics under rolling and flipping (*SI Appendix*).

## Supplementary Material

Supplementary File

## Data Availability

All study data are included in the article and/or *SI Appendix*.

## Change History

March 19, 2021: The license for this article has been updated.
