# Supplementary material for: Bistability in oxidative stress response determines the migration behavior of phytoplankton in turbulence
Source: Proc Natl Acad Sci U S A. 2021 Jan 25;118(5):e2005944118. doi: 10.1073/pnas.2005944118 (PMC7865155; doi:10.1073/pnas.2005944118)
Supplement: Supplementary File [file pnas.2005944118.sapp.pdf]

PNAS
www.pnas.org

Supplementary Information for
Bistability in oxidative stress response determines the migration behavior of phytoplankton in turbulence

Francesco Carrara[1#], Anupam Sengupta[1,2*#], Lars Behrendt[1,4], Assaf Vardi[3] and Roman Stocker[1*]

1. Institute for Environmental Engineering, Department of Civil, Environmental and Geomatic Engineering, ETH Zurich, 8093 Zurich, Switzerland
2. Department of Physics and Materials Science, University of Luxembourg, 162 A, Avenue de la Faïencerie, 1511, Luxembourg
3. Department of Plant and Environmental Sciences, Weizmann Institute of Science, Rehovot 7610001, Israel
4. Science for Life Laboratory, Department of Environmental Toxicology, Uppsala University, Norbyv. 18A, 75236 Uppsala, Sweden

#Contributed equally to this work
*Email: anupam.sengupta@uni.lu; romanstocker@ethz.ch

This PDF file includes:

Materials and Methods
Supplementary Text
Figures S1 to S12
Tables S1 to S2
References S1 to S20

Materials and Methods

Behavioral and stress response of *Heterosigma akashiwo*. Here we summarize the sequence of events involved in our experiments probing the behavioral and stress response of *Heterosigma akashiwo* upon exposure to turbulent cues:

- Pre-treatment, most cells in the control (on average 77.5%) swim upwards (average upward bias $r = 0.55$ from Fig. 1; see **Upward bias index** for details) as a result of the torque produced by their mechanical stability (negative gravitaxis). We quantified the cells' mechanical stability at the single-cell level using time-lapse microscopy (Fig. 1E) (see **Quantification of the cell stability parameter** for details.)
- Post-treatment (flipping experiments from Fig. 1), on average 54.5% of cells continue to swim upwards, whereas the remaining 45.5% of cells are observed to swim downwards (see **Generation of turbulent cues**). There is thus a behavioral split in the population to form two subpopulations of cells with opposite mechanical stability (performing negative or positive gravitaxis). The change in mechanical stability is underpinned by a rapid morphological change that modulates the torque balance within a cell (*S1*).
- We quantify this split (how many cells swim upward, how many swim downward) by (i) waiting for 30 min after the treatment ceased, so that upward-swimming cells ended up near the top of the chamber and downward-swimming cells ended up near the bottom of the chamber and then (ii) used time-lapse microscopy to count cells at the top and bottom of the chamber and to infer their mechanical stability (see **Cell tracking**).
- We collected cells from the top and bottom for stress analysis and analyzed those cells by flow cytometry after exposure to rolling experiments (see **Flow cytometry data analysis**), which produce an equivalent flow pattern and induce a similar behavioral response to those generated by flipping. We compared the stress levels (measured in the form of an increase in ROS) between top and bottom subpopulations and with the control cells, stained through the same procedure. We also measured the stress dissipation timescale after exposure to rolling (see **Recovery after endogenous stress**).
- This revealed that the subpopulation that actively changed its direction of migration and accumulated at the bottom of the experimental container showed a two-fold increase in ROS compared to the subpopulation at the top of the container.
- Separately, we conducted experiments with ROS scavengers and showed that we could block the behavioral response. We could also mimic the behavioral response and the bistable stress response by applying external ROS (in the form of H_2O_2 and high irradiance) that activated the response downstream in the signaling cascade (see **Exogenous H_2O_2 exposure experiments** and **Upward bias induced by near UV-A exposure and by strong irradiance**).

Generation of turbulent cues. Two different experimental modes – *flipping* and *rolling* – were employed to generate turbulent cues for the experiments reported here. The flipping mode, carried out in a flip chamber, was optimal for visualizing swimming cells and their distribution in the vertical plane, whereas the rolling mode was well suited for experiments that required larger cell culture volumes, such as those requiring subsequent fluorescent analyses using flow cytometry. Both experimental modes produced equivalent turbulent cues and yielded no difference in the turbulence-induced phytoplankton response (Fig. S4). While the rolling mode consisted of a continuous reorientation of the confined suspension in one direction, in the flipping mode, the direction alternated between clockwise and counterclockwise. The turbulent signal can be decomposed into triangular waves for reorientation occurring over different rotation time, τ_R,

and square waves for different resting times between reorientation, τ_W (Fig. 1A,B). In our experiments, for instance, periodic flipping consisting of multiple, rapid overturning of the chamber (180° in 3 s; τ_R = 3 s), each followed by 15 s at rest (τ_W = 15 s), results in a period of 18 s. This corresponds to the Kolmogorov timescale $\tau_K = (v/\varepsilon)^{1/2}$, where v is the kinematic viscosity of the fluid (seawater), and ε is the turbulent energy dissipation rate. The turbulent kinetic energy dissipation associated with τ_W = 15 s and τ_R = 3 s is $\varepsilon = 3 \times 10^{-8}$ W kg^{-1}, a value typical of the ocean pycnocline, falling within the typical range of values for ocean turbulence (10^{-10}–10^{-5} W kg^{-1}) (*S2, S3*). Similarly, the turbulent kinetic energy dissipation corresponding to τ_W = 0 s and τ_R = 3 s is $\varepsilon = 10^{-7}$ W kg^{-1}. The two different generators of turbulent cues are described below.

(i) Flipping. A millifluidic flip chamber (12 mm × 4 mm × 1.6 mm) made of transparent acrylic was used to visualize the motility and upward bias of *H. akashiwo* cells. The flip chamber was mounted to the shaft of a stepper motor that allowed for full reorientations from 0° to 360°. For the flipping experiments, the rotation of the chamber was automated using an externally programmed controller that drove the motor, with full user control over the time series of the rotation angle. For all experiments (including experiments with cells exposed to exogenous H_2O_2, UV-A, strong radiation, and after treatment with ROS scavenger KI), a 75 µl suspension of phytoplankton cells was gently pipetted into the chamber through one of two injection ports, which were then closed with silicone plugs. During experiments, cells in the flipping chamber were visualized using a stereoscope (Nikon SMZ1000) with a Plan APO ×1 objective (0.12 NA) and a digital CMOS camera (Photron FastCam SA3). The flipping chamber was mounted on a translation stage, the position of which could be controlled using micrometer screws along all three axes. The camera was focused on a plane perpendicular to the rotation axis and midway between the two chamber walls. The depth of focus was 750 µm, ensuring that cells were more than 400 µm (>50 cell radii) from the front and back walls of the chamber, to eliminate wall effects. Any small residual wall effects that may still have occurred would have been present for the entire duration of an experiment, and thus could not have caused the population split. Images were acquired at 60 frames per second. The suspension was uniformly illuminated using a single 627 nm LED (SuperBright LEDs, RL5-R12008, 0.1 W) mounted just outside of the flipping chamber. Neither of the two *H. akashiwo* strains tested showed any phototactic bias to wavelengths of light in the red spectrum, in agreement with literature (*S4*). All experiments were conducted under diffused room light settings, to avoid possible photo-responses. For each treatment, a control experiment was performed in which cells were observed in the flipping chamber without rotation, for the same duration as the treatment. The vertical distribution of cells in these control experiments was quantified at regular intervals to confirm that the upward bias of cells in the absence of overturning remained constant.

(ii) Rolling. Turbulence experiments for subsequent flow cytometry and microscopy-PAM measurements (see below) were carried out using a programmable rolling device (Thermo Fischer Scientific Tube Roller). Cell suspensions were pipetted into cylindrical glass vials of 2 ml maximum volume, filling them to capacity to leave no empty space. The glass vials, placed horizontally on the tube roller, underwent rolling for a pre-assigned duration at a desired angular speed. The turbulence energy dissipation rates of the rolling experiments at 10 rev min^{-1} (Ω = 1 rad s^{-1}) quantitatively matched those in the flipping experiments at a rotation time of τ_R = 3 s (Fig. S4). Post rolling, the cylindrical vial was left undisturbed for 30 min in the vertical position, so that the swimming cells could attain their stationary population distribution. The top and bottom subpopulations were then harvested for subsequent analyses and physiological experiments. As in the case of the flip experiments, all rolling experiments were conducted under diffused room light settings, and for each treatment, a control experiment was performed in which cells were observed in a cylindrical vial without rolling for the same duration as the treatment. The vertical distribution of cells in these control experiments was quantified at regular intervals to confirm that the upward bias of cells in the absence of rolling remained constant.

Upward bias index. After the end of every flipping experiment, we allowed the population to reach its equilibrium distribution over the vertical driven by the migration behavior by waiting 30 min. This period was chosen conservatively based on the observation that the concentration

profile already stabilized after ≈5 min, and the consideration that a cell migrating upward at v = 88 μm s^{-1} (the mean swimming velocity, Fig. S12A) would cover the depth of the flipping chamber (4 mm) in less than 2 min. To quantify the migration behavior of the cells, we first obtained histograms of normalized cell concentration in the flipping chamber, within the region captured by the camera (4 mm × 4 mm) in the mid-chamber plane of focus. To quantify the asymmetry in cell distribution over the vertical, we computed the upward bias $r = (f_{\uparrow} - f_{\downarrow})/(f_{\uparrow} + f_{\downarrow})$, where $f_{\uparrow}$ and $f_{\downarrow}$ are the numbers of cells in the top 400 μm and the bottom 400 μm of the chamber, respectively (following the method developed in ref. *S1*). We decided to adopt the upward bias index, as opposed for example to the percentage of upward-swimming cells, based on the intuitive association of the sign of the bias with the average direction of migration of a population of cells. Positive and negative values of the upward bias r indicate upward and downward migrating populations, respectively. We note that both the upward bias and the percentage of upward-swimming cells are linear metrics, which range from −1 to +1 and from 0% to 100%, respectively. A symmetric distribution of cells corresponds to $r = 0$, whereas preferential upward-migrating corresponds to $r > 0$ and preferential downward-migrating to $r < 0$. After the flipping experiments, the two subpopulations of *H. akashiwo* migrating upward (↑) and downward (↓) were used in measurements of the mechanical stability (Fig. 1C). Control experiments consisted of cells held in the chamber for the entire duration of the flipping experiments without flipping.

Cell tracking. To extract the swimming behavior of cells (swimming speed and stability), movies were recorded at 12 frames per second. For tracking, cell locations were determined by image analysis based on intensity thresholding using MATLAB (MathWorks) routines (*S5*). Cell trajectories were assembled by linking the locations of cells in subsequent frames, based on proximity and kinematic predictions from previous time steps, using automated software. Cells in the flipping chamber swam in helical patterns, characteristic of many motile phytoplankton species (*S6*). However, the helical component was averaged out using a 1-s moving average to reduce noise in the calculation of the stability parameter A.

Quantification of the cell stability parameter. To determine cell stability at the population level for the *H. akashiwo* CCMP452 and CCMP3374 strains, we quantified the rotation rate ω of cells as a function of their orientation θ relative to the vertical. This is an established method for quantifying the reorientation timescale B, as greater stability will cause faster reorientation towards the stable orientation after a cell is perturbed. The greater the magnitude of B, the lower the mechanical stability, with the sign of B denoting the upward ($B > 0$) or downward ($B < 0$) stable swimming direction. The stability parameter is then calculated as $A = (2B)^{-1}$ (ref. *S7*). To this end, we tracked individual cells over 15 s immediately following a single flip (which provided the perturbation from the stable orientation), and averaged their rotation rate over all cells as a function of θ. The resulting data for $\omega(\theta)$ were fitted well by a sinusoidal function of the form $a \sin(\theta + \kappa)$, with a the amplitude of the sinusoid, and where we imposed a phase shift κ equal to π for the upward-migrating cells (simultaneously fitting both a and κ showed consistent results for this approach). We determined the reorientation timescale (Fig. S1A) from the best-fit sinusoid as $B = (2a)^{-1} \sin \kappa$. To account for heterogeneity in the stability, which would result in some cells reorienting faster than others, we also quantified the stability parameter A for CCMP452 cells at the single-cell level. From among the trajectories analyzed to extract the population stability parameter, we selected those that had data points with the orientation θ between $-\pi/3$ and $\pi/3$. This allowed us to extract the stability parameter from the trajectory in the approximately linear region of the sinusoidal dependence. Using this approach, we determined the distribution of the parameter A for cell populations from each of the different treatments (control, and following N = 30, 100, 300 flips for cells from the top and bottom subpopulations; Fig. 1E).

Flow cytometry data analysis. *H. akashiwo* cells were identified by gating a suitable region in the 2D scatter plot using the channels APC-A (allophycocyanin, red fluorescence at 763/43 nm) vs. FSC-A (forward scatter, correlating with cell size, ref. *S8*). This gating was kept fixed for all subsequent experiments and ensured that only viable (i.e., red autofluorescing) *H. akashiwo* cells of a certain size were included in subsequent analyses (thereby excluding bacteria and other debris). Using the above gating strategy, FITC-A fluorescence was recorded across different

treatments and the mean values of the different replicates extracted. The standard deviation associated with the measurements represents the variation of the mean values of the FITC-A channel across different replicates.

Recovery after endogenous stress. To quantify the ability of *H. akashiwo* to recover from stress, four replicates were extracted from the same culture, introduced to 2 ml glass vials, and exposed simultaneously to 5 min of reorientations on the tube roller at 10 rev min^{-1} (equivalent to a fast rotation timescale of τ_R = 3 s, which is equivalent to a rotation rate, Ω = 1 rad s^{-1}). After the rolling stopped, CM-H_2DCFDA was added at a final concentration of 10 μM. The oxidative marker was introduced at four different time points, t = {30, 90, 210, 630} s, in the four different populations. All samples were incubated under dark conditions for 30 min and subsequently FITC-A fluorescence intensities were quantified using a flow cytometer. An exponential curve was fitted to the mean values of the replicates, weighted by their standard deviation, to obtain the stress dissipation timescale τ_S.

Exogenous H_2O_2 exposure experiments. Hydrogen peroxide (H_2O_2), a common oxidative species, was used to study the behavioral response of *H. akashiwo* to an exogenous oxidative stressor. Hydrogen peroxide solution (30% (w/w) in H_2O, Sigma-Aldrich), was dissolved in the CCMP452 cell suspension to achieve final concentrations spanning four orders of magnitude, from 1 μM to 1 mM. Cells were incubated in either 0 (control), 1, 3, 10, 15, 23, 33, 100 μM or 1 mM H_2O_2 for 15 min. The cells were then carefully transferred to the flip chamber and allowed an additional 15 min to reach their stationary swimming distribution. Using the imaging protocol described above (see "**Generation of turbulent cues: *(i)* Flipping**"), the upward bias was calculated from the equilibrium vertical distribution.

Upward bias induced by near UV-A exposure and by strong irradiance. *H. akashiwo* cells were carefully pipetted into 24-well plates (Corning inc., Corning, NY, USA) using an enlarged (i.e., cut) pipette tip. The filling volume (1 ml) was chosen to achieve a maximum depth of 5 mm to avoid self-shading effects within the cell suspension. The 24-well plate was placed onto black masking tape (Thorlabs, Newton, NJ, USA) to avoid back scattering and irradiance was provided to a single well by a collimation lens (Thorlabs) connected to a light source via a liquid light guide. For illumination, two different light sources were used, (*i*) an LED-based Lumencor Spectra X light engine (Lumencor, Beaverton, OR, US) for targeted irradiance with UV-A (380–400 nm), and (*ii*) a mercury-based Intensilight light source (Nikon Corp., Tokyo, Japan) for full spectrum irradiance (320–800 nm, peak wavelengths at 407 nm, 429 nm, 534 nm and 575 nm). Different levels of photon quanta were achieved by the automated insertion of neutral density filters (Intensilight) or the regulation of voltage (Spectra X light engine). Full spectrum irradiance was calibrated via a cosine-corrected mini quantum sensor (MQS-B, Walz) placed at the level of the top of the culture suspension and connected to a light meter (ULM-500, Walz). In a similar fashion, near UV-A irradiance levels were quantified using a GigaHertz BTS256-EF luxmeter (Gigahertz-optics GmbH, Türkenfeld, Germany), using a spectral cut-off matching that of the light source (380–415 nm) and by exporting both photon flux densities in units of μmol m^2 s^{-1} and unweighted UV-A irradiances. Displayed photon flux densities (20, 78 and 116 μmol m^2 s^{-1}, Fig. 2E) correspond to unweighted UV-A irradiances of 16, 61 and 85 W/m^2, respectively. Cells within 24-well plates were exposed for 30 minutes to near UV-A of different intensities and following exposure, cells were carefully transferred to the millifluidic flip chamber and their upward bias quantified using the methods described above. Corresponding control cells were placed in the same 24-well plates, but covered with a dark foil to avoid exposure to UV-A.

Statistical analysis. We performed a one-way ANOVA to compare the upward bias index r among the still control and cells from two populations having undergone reorientations: cells grown in the presence of scavenger potassium iodide, and cells grown under the standard conditions. We made multiple comparisons using a post-hoc Tukey's HSD test (Table S1). The same statistical analysis was conducted to compare the maximum quantum yields among the still control and cells from top and bottom subpopulations after the exposure to turbulent cues (Table S2). Experiments were performed with at least three replicates, with the number of replicates for

each experiment reported in figure captions. All the replicates in our experiments were biological replicates.

Supplementary Text

Stress dynamics of gravitactic cells under turbulent cues. An effect of rotation is that the gravitational acceleration g appears to be rotating in the rotating frame of reference, i.e., for cells that tumble and perform periodic orbits because of a low mechanical stability. These changes in the cell orientation relative to gravity may cause the organism to behaviorally respond by switching the migratory direction from negative to positive gravitaxis, where the cue for the behavioral differentiation is not the direct effect of fluid velocity gradients (e.g., shear), but rather the eddies inducing changes in the cell orientation relative to gravity (*S1*). Above some critical value of Ω_c at which $|\Omega_c| > |A|$, the cell tumbles by fluid shear in the vertical plane (Fig. S1C). For simplicity's sake, we assume that before a tumbling event induced by flipping or rolling, the cell swims at its equilibrium position, that is, for negatively gravitactic cells such as *H. akashiwo*, the direction opposite to gravity, at which the angle $\theta = 0$ relative to gravity. We can write the force-free condition along this direction of swimming (but a similar argument holds for any swimming direction) as $P + B - G - D_{\uparrow} = 0$, where P is the propulsion force from the beating of the flagellum, $B = \rho_f V g$ and $G = \rho_p V g$ are the buoyancy and gravity forces acting on the cell respectively with V the volume of the cell, ρ_f and ρ_p the densities of the fluid and of the cell, g is the gravity acceleration, and $D_{\uparrow}$ is the drag force directed in the opposite direction to the cell swimming velocity when the cell's swimming is at the angle $\theta = 0$ relative to gravity. After a tumbling event, for example induced by flipping or rolling, the cell's swimming velocity is directed at an angle $\theta = \pi$ relative to gravity. At the downward-swimming orientation, the force-free condition is $-P + B - G + D_{\downarrow} = 0$, where the propulsion P and the drag force $D_{\downarrow}$ switched sign as a consequence of the change in the swimming direction relative to gravity due to the reorientation. If we assume that the propulsion does not change as a consequence of the tumbling event, by combining the two conditions it gives the difference in the drag force experienced by the cell, $F_g = D_{\downarrow} - D_{\uparrow} = 2\,(G - B)$. Generalizing to any direction θ relative to gravity upon a tumbling event, a cell is therefore subjected to an impulsive force of magnitude $F_g = (\rho_p - \rho_f)\, gV\,(1 - \cos\theta)$. For *H. akashiwo,* which is characterized by an excess density $(\rho_p - \rho_f) \sim 50$ Kg m^{-3} and an equivalent radius $R \sim 7$ µm (*S1*, *S9*, *S10*), the impulsive force generated by the gravity force during a tumbling event is $F_g = 1.5$ pN at $\theta = \pi$. The value for F_g is considerably larger than the force exerted by fluid shear $F_{shear} = v\rho_f S\, \partial u/\partial z$ (ref. *S3*), where $v = 10^{-6}$ m^2 s^{-1} is the kinematic viscosity of seawater, S is the surface of the cell, and $\partial u/\partial z$ is the shear experienced by the cell under turbulence. For example, at a turbulence dissipation rate $\varepsilon = 10^{-8}$ W kg^{-1}, cells are subjected to an average shear $\partial u/\partial z \sim 0.1$ s^{-1}, at which $F_{shear} = 0.06$ pN, which is 25 smaller than F_g. The two forces F_g and F_{shear} have equivalent magnitude for very high levels of ocean turbulence, $\varepsilon = 7 \times 10^{-6}$ W kg^{-1} (*S2*, *S3*), which are never reached in our reorientations experiments. In our model of stress dynamics, we therefore focus on the effects of the impulsive force F_g on the ROS accumulation-dissipation dynamics, and we neglect the small contribution from the shear force. We hypothesize that during a tumbling event intracellular stress is generated in the cell under the form of a nearly instantaneous release (*i.e.*, a spike) of ROS whenever the cell is being reoriented relatively to gravity, that is, when the cell experiences a force of typical magnitude F_g. In our model, the ROS spike specifically occurs at times t_i whenever the cell swims in a direction $\theta = \pi/2$, that is in the direction perpendicular to the gravity vector. This particular choice of swimming direction is arbitrary, and we could choose any value between $\pi/2 < \theta < \pi$ without changing our results. The intracellular scavenging machinery of the cells dissipates the accumulated stress s with a characteristic timescale, τ_S (measured experimentally, Fig. 3B; see "**Recovery after endogenous stress**" above). By way of example, the scavenging machinery in the raphidophyte *Chattonella marina* – a harmful-algal-bloom species which exhibited a migratory response to turbulence similarly to *H. akashiwo* (*S1*) – comprises several antioxidant enzymes, including glutathione peroxidase, peroxiredoxin, catalase, and ascorbate peroxidase (*S11*). In principle, the characteristic stress dissipation timescale τ_S depends on the cellular antioxidant capacity: compare the response to flipping for two populations with and without the additional scavenging supplement of KI (Fig. 2F). However, all the turbulence experiments were performed with cells

grown under the same conditions at a fixed period of the day to avoid diurnal fluctuations in population physiology (*S12*), and we do not expect the antioxidant capacity to vary over the relatively short experimental timescales (<20 min rolling, Fig. 2A). We therefore assumed the stress dissipation timescale τ_S as a constant parameter in our model. The resulting intracellular stress accumulation–dissipation dynamics are captured by the following differential equation

$$ds/dt = \sum_{t_i} \Delta s_i \, \delta(t - t_i) - s\tau_S^{-1} + c_0 \, , \qquad \text{Eq. S1}$$

where the Dirac delta function $\delta(t - t_i)$ records the stress spikes Δs_i (assumed to all have the same value Δs) occurring at times t_i for a given swimming trajectory, and c_0 is the baseline stress rate. Eq. S1 can be solved by performing the Laplace transform, which gives the stress level as a function of time

$$s(t) = \sum_{t_i} \Delta s \, \theta(t - t_i) \, e^{-(t-t_i)\tau_S^{-1}} + s_0 \, , \qquad \text{Eq. S2}$$

where $\theta(t - t_i)$ is the Heaviside function, and $s_0 = c_0 \, \tau_S$ is the baseline stress level before the fluid rotation.

In the following paragraphs, we model the stress dynamics for cells exposed to turbulent cues for the two paradigmatic cases that we employed experimentally: *i*) a continuous solid body rotation (i.e., rolling) with rotation rate $\Omega = \pi/\tau_R$ (no resting phases, $\tau_W = 0$), and *ii*) multiple, fast reorientations of amplitude π (i.e., flipping) occurring at a rate $\Omega >> A$, alternating with resting phases captured by the timescale τ_W (Fig. 1B).

(i) ***Cell stress generated by rolling.*** For a population of cells of *H. akashiwo* CCMP452 swimming in a rotating chamber at a constant rotation rate $\Omega = \pi/\tau_R$, where τ_R is the time taken for half a revolution (that is, the rotation time parameter in the flipping), a fraction of cells from the population distribution (see **Quantification of the cell stability parameter** above) has a stability parameter *A* (high mechanical stability) higher than the rotation rate Ω, such that these cells swim at a certain angle θ_{eq}. For this fraction of cells, the continuous rotation does not induce any additional stress above the baseline levels. The remaining cells, which tumble under the continuous rotation, accumulate stress over time at each rotation according to the stress model (Eq. S2). The direction $\theta(t)$ of a gravitactic cell characterized by stability parameter *A,* exposed to a constant rotation rate Ω (that is, a vortical flow with vorticity ω = 2Ω, analyzed in Pedley & Kessler (*S13*), is

$$\theta(t) = 2\arctan\left[A + \sqrt{\Omega^2 - A^2}\tan\left[\frac{t}{2}\sqrt{\Omega^2 - A^2} - \arctan\left[\frac{A}{\sqrt{\Omega^2 - A^2}}\right]\right]\right], \qquad \text{Eq. S3}$$

with initial conditions $\theta(t = 0) = 0$ and provided that $|\Omega| > |A|$. The period of the trajectory for a tumbling cell is set by the period in the argument of the tan function. For the solution reported above, which is the solution used to plot the curves in Fig. S1C, the period is equal to $T_B = 2\pi\,(\Omega^2 - A^2)^{-1/2}$. This result follows by considering that the period of tan[$x+a$] is π, where $x = ½\, t\,(\Omega^2 - A^2)^{1/2}$ and $a = \arctan[A(\Omega^2 - A^2)^{-1/2}]$ is a constant. The solution for the tumbling period has a divergence at $|\Omega| = |A|$, where Eq. S3 characterizing the swimming angle θ has a standard bifurcation. By taking the partial sum in the summation in Eq. S2, the upper envelope of the stress signal over time experienced by the tumbling cells is

$$s_{max}(t) = \Delta s\frac{\left(1 - e^{-N\,T_B\tau_S^{-1}}\right)}{\left(1 - e^{-\,T_B\tau_S^{-1}}\right)} + s_0 \, , \qquad \text{Eq. S4}$$

where the sequence of times t_i at which stress is generated is $S = \{T_B, \ldots iT_B, \ldots, NT_B\}$ after *N* periodic reorientations, and T_B is the period of the orbit for the tumbling cells (*S13*), which depends on the stability timescale $B = (2A)^{-1}$ and on the rotation rate Ω. Note that for $A = (2B)^{-1} << |\Omega|$, a cell's trajectory becomes almost circular with period $T_B \xrightarrow[B^{-1} \ll 2\Omega]{} 2\tau_R = 2\pi/|\Omega|$ (Fig. S1B).

For values of rolling of Ω = 1 rad s^{-1} implemented experimentally in Figs. 3,4 and the stability parameter A = 0.09 s of *H. akashiwo* CCMP452 (Fig. S1A), the period of a cell's trajectory T_B differs from the period of chamber rolling $2\pi/\Omega$ only by 1%. After multiple reorientations, N >>1, the numerator of the first term on the right hand side of Eq. S4 converges to Δs, and the expression is well approximated by

$$s_{max} \sim \Delta s \left(1 - e^{-T_B \tau_S^{-1}}\right)^{-1} + s_0 \,, \qquad \text{Eq. S5}$$

where $T_B = 2\tau_R$. Under these conditions, the cells with low mechanical stability and/or low antioxidant capacity, by accumulating a relative stress level s_{max}/s_0 exceeding the stress threshold h, switch the direction of migration from upward to downward. The values of s_{max} as a function of the tumbling period $T_B = 2\pi\,(\Omega^2 - A^2)^{-1/2}$ calculated from Eq. S5 predict the experimental data for the stress levels presented in Fig. 2B as a function of the rotation rate Ω. We used the stress dissipation timescale τ_S = 87 s from the experiments presented in Fig. 3B, and we fitted the value of $\Delta s = 0.4\ s_0$ through the least squares method (Fig. 3D, black line). The value of the tumbling period T_B = 32 ± 13 s (which is equivalent to the resting time for flipping, compare Eq. S5 and Eq. S8) for which $s_{max} = h$ calculated from Eq. S5 matches the resting time τ_W = 40 s at which cells perform the migratory switch in the experiments presented in Fig. 1F, with stress dissipation timescale τ_S = 87 s from the experiments presented in Fig. 3B, and $\Delta s = 0.4\ s_0$, which is the free parameter fitted by stress data presented in Fig. 3D. We also used the same value of Δs to predict the stress distribution after rolling for the top and bottom subpopulations (Fig. S10) (see **Stochastic analysis of stress dynamics of gravitactic phytoplankton navigating in turbulent flows** below).

(*ii*) *Cell stress generated by flipping.* In the case of flipping the chamber at a fast rotation rate $\Omega_{max} = \pi$ rad s^{-1} (τ_R = 1 s), the condition $A_{max}|\Omega_{max}| > 1$ is satisfied, where A_{max} = 1.1 rad s^{-1} is the highest mechanical stability parameter of the population (Fig. 1E). Under these conditions, which corresponds to the case tested experimentally (Fig. 1F), the entirety of the cells in the population would tumble during the flip. Compared to the (slower) continuous rotation case (Fig. 1D), where the emerging downward-migrating is mediated by the distribution of mechanical stabilities in the population, the dynamics for the upward bias present a more pronounced threshold as a function of the resting phase τ_W because all the cells under this experimental configuration accumulate stress at each flip (Fig. 1F). The accumulation–dissipation dynamics of cellular stress are set by the ratio of the three timescales of the process, the dissipation timescale τ_S and the period of time between two flips $\tau_W + \tau_R$ (the sum of the resting phase between two flips and the time taken to flip the chamber). We can obtain the maximum stress over time for a cell under multiple periodic reorientations (flips) as

$$s_{max}\,(t) = \Delta s \frac{\left(1 - e^{-N\,(\tau_W + \tau_R)\tau_S^{-1}}\right)}{\left(1 - e^{-(\tau_W + \tau_R)\tau_S^{-1}}\right)} + s_0 \,. \qquad \text{Eq. S6}$$

The stress accumulation–dissipation dynamics captured by Eqs. 4 and 5 are plotted in Fig. 3C for the different values of τ_W, which correspond to the range of experimental values for the resting times (Fig. 1F). After many (100) flips, N >>1, the numerator of the first term on the right hand side of Eq. S6 converges to Δs, and the expression is well approximated by

$$s_{max} \sim \Delta s\left(1 - e^{-(\tau_W + \tau_R)\tau_S^{-1}}\right)^{-1} + s_0 \,. \qquad \text{Eq. S7}$$

Provided that in our experiments $\tau_R << \tau_W$ (except for the cases of continuous flipping or rolling with τ_W = 0 s, which are captured by Eqs. S4 and S5), the expression in Eq. S7 can be further simplified as

$$s_{max} \sim \Delta s\left(1 - e^{-\tau_W/\tau_S}\right)^{-1} + s_0 \,. \qquad \text{Eq. S8}$$

where for $\tau_S/\tau_W >> 1$ the first term on the right side becomes much greater than one after rescaling by s_0, and therefore greater than the stress threshold $h = 2.3$ for the migratory switch (extracted from results presented in Fig. S8A). The value of the resting time $\tau_W = 32 \pm 13$ s for which $s_{max} = h$ calculated from Eq. S8 matches the resting time $\tau_W = 40$ s at which cells perform the migratory switch in the experiments presented in Fig. 1F (with parameters $N = 100$ flips, the stress dissipation timescale $\tau_S = 87$ s from the experiments presented in Fig. 3B, and $\Delta s = 0.4\ s_0$, which is the free parameter fitted by stress data presented in Fig. 3D). In the section below "**Stochastic analysis of stress dynamics of gravitactic phytoplankton navigating in turbulent flows"**, we derive the approximation for s_{max} in the limit $(\tau_W\, \tau_S^{-1}) \to 0$ where the term in the parenthesis diverges, which results in a hyperbolic increase as a function of the ratio $z = \tau_W\, \tau_S^{-1}$.

Phytoplankton navigation in isotropic turbulence. In principle, Eq. S2 is applicable to describe the stress dynamics of cells navigating in any turbulent field (Fig. S2A). The validation of the model on a simplified representation of intermittent turbulence allows its use to investigate the migratory and physiological performance of cells in three-dimensional isotropic turbulent flows (*S14*), for example through direct numerical simulations (*S15, S16*) or turbulence tank experiments (*S17-S19*). Importantly, for a given turbulent signal, the cell's mechanical stability affects the distribution of the tumbling (Fig. S2B) and resting phases (Fig. S2C), and ultimately determines the migratory behavior of a cell (Fig. 3C). In analogy to the simple case of a gravitactic cell swimming under continuous rolling at a rotation rate Ω considered in Eq. 2 in the main text, where the period T_B sets the tumbling and therefore the resting time statistics, the characteristic resting time under turbulence is a function $\tau_W(\varepsilon, A)$ of the turbulent kinetic energy ε, which sets the Kolmogorov timescale τ_K of the microscale turbulent eddies, and of the stability parameter, A. To determine the time series of the rotation rate relative to the vertical, $\Omega(t)$, shown in Fig. 3A, we used the time history of the angular orientation of a small passive sphere in homogeneous isotropic turbulence (*S15*), quantified from a direct numerical simulation at $Re_\lambda = 65$ (time history courtesy of M. Cencini and G. Boffetta), where $Re_\lambda = u_{RMS}\, \lambda/\nu$ is the Taylor Reynolds number, which represents the range of length scales characteristic of a given turbulent flow, with $\lambda = u_{RMS}\, (15\nu/\varepsilon)^{1/2}$ the Taylor length scale, u_{RMS} the root-mean-square fluid velocity, and ε the turbulent energy dissipation rate.

Stochastic analysis of stress dynamics of gravitactic phytoplankton navigating in turbulent flows.

For $|\Omega| > |A|$, the angle of a swimming cell (see Eq. S3) will continuously drift in one direction at a very inhomogeneous rate with period given by $T_B = 2\pi\, (\Omega^2 - A^2)^{-1/2}$. However, for the rotation rate $\Omega = 1$ rad s^{-1} considered in the experiments reported in Fig. 2A, there is practically no difference (<1%) in the period of the trajectory for tumbling cells for 95% of cells, based on the distribution of the stability parameter observed in the population (Fig. S9A). The distribution of stability parameters satisfies the condition $A << \Omega$, where the leading order term for the tumbling period is $T_B \approx 2\pi\Omega^{-1}(1 + ½\, (A/\Omega)^2\,)$. The tumbling period appears in the expression of the stress $s_{max}(t) = \Delta s \frac{\left(1 - e^{-N\, T_B \tau_S^{-1}}\right)}{\left(1 - e^{-T_B \tau_S^{-1}}\right)} + s_0$, so that the steady-state solution of the stress after many ($N >> 1$) flips reads $s_{max} \sim \Delta s \left(1 - e^{-T_B \tau_S^{-1}}\right)^{-1} + s_0$, where s_0 is the baseline stress level and τ_S is the stress dissipation timescale. Under the experimental conditions of Fig.2A, with rotation rate $\Omega = 1$ rad s^{-1}, the tumbling period is $T_B \approx 2\pi << \tau_S = 87$ s, and the term in the parenthesis in the expression for s_{max} diverges for $(T_B\, \tau_S^{-1}) \to 0$.

By introducing the complex variable $z = T_B\, \tau_S^{-1}$, we develop the first three nonzero terms of the Laurent expansion of the function $f(z) = (1 - e^{-z})^{-1}$ about the origin. Note that the function is analytic on the annulus $0 < |z| < 2\pi$. Since $1/f(z)$ has a zero of order 1 in $z = 0$, $f(z)$ has a simple pole in $z = 0$, so the Laurent expansion reads $f(z) = a_{-1}/z + a_0 + a_1 z + \ldots$ for all values of z in this annulus. Multiplying both sides by $1 - e^{-z}$, we obtain $1 = (a_{-1}/z + a_0 + a_1 z + O(z^2))\, (-z + z^2/2! - z^3/3! +$

$O(z^4)$), where we used the Taylor expansion of the function e^{-z}. By comparing coefficients with the same power we obtain for the first three non-zero coefficients $a_{-1} = 1$, $a_0 = ½$, and $a_1 = 1/12$. The approximation for $f(z)$ thus reads $f(z) = 1/z + 1/2 + 1/12\ z + O(z^2)$, with $z = T_B\ \tau_S^{-1}$. The expression for s_{max} above becomes

$$s_{max} = \Delta s\ (1/2 + T_B^{-1}\tau_S + 1/12\ T_B\tau_S^{-1} + O((T_B\ \tau_S^{-1})^2)) + s_0\,. \qquad \text{Eq. S9}$$

Since we are interested in the solution in the neighborhood of the origin, $z = 0$, where $T_B < \tau_S$, we further simplify the expression for the stress s_{max} in Eq. S9 by dropping the linear term of the Laurent expansion without affecting the accuracy of the solution. By substituting the approximation for the tumbling frequency $1/T_B$ (see Fig. S9A)

$$1/T_B \approx 2\pi\Omega^{-1}[1 - ½(A/\Omega)^2], \qquad \text{Eq. S10}$$

we obtain the following expression for the stress at steady state (see Fig. S9B, where we plot s_{max} rescaled by s_0)

$$s_{max} \approx \Delta s\ [(2\pi)^{-1}\ \Omega\ (1 - ½\ (A/\Omega)^2)\ \tau_S + 1/2] + s_0\,. \qquad \text{Eq. S11}$$

By means of the expression derived in Eq. S11, we can compute the *pdf* for the stress at steady state. Based on the experimental distribution of the stability parameter reported in Fig. 1E, we model the distribution of the stability parameter as a Gaussian distribution $F(A;\ \mu, \sigma)$, with mean $\mu = 0.10$ rad s^{-1} and standard deviation $\sigma = 0.21$ rad s^{-1} (Fig. S9C). We invert Eq. S11 to find the expression

$$A_{\pm}(s,\tau_s) = \pm\sqrt{\frac{\Omega(2\pi\Delta s - 4\pi s + 4\pi s_0 + 2\Delta s\tau_s\Omega)}{\Delta s\tau_s}}, \qquad \text{Eq. S12}$$

where $A_{\pm}$ are the positive and negative branches of the solution for the stability parameter corresponding to upward- and downward-swimming cells, respectively. By applying the transform rule for random variables, the distribution of stress in a population of *H. akashiwo* whose cells have variable stability parameters can be expressed as

$$H(s) = \sum_{\pm} F(A_{\pm}(s, \tau_s);\ \mu, \sigma)\left|\frac{dA_{\pm}}{ds}\right|, \qquad \text{Eq. S13}$$

where the term $\sum_{\pm} F(A_{\pm}(s, \tau_s);\ \mu, \sigma)$ sums over the two branches of the *pdf*s for the stability parameter.

We can also derive the exact numerical expression for the stress without the approximation in Eqs. S10-S11, implemented for the function $f(z)$ and for the period T_B. We found very good agreement with our approximated solution for $H(s)$ across different values of the dissipation timescales and stability parameters aimed to model the different strains and subpopulations of *H. akashiwo* (Fig. S9D). In fact, we derived the *pdf* of the stress for strain CCMP3374, which has a higher stability parameter. These results show very little effect induced by the variation in the stability parameter on the *pdf* of the steady state in both strains CCMP452 and CCMP33374, which is the consequence of two factors: i) the small differences in the periods of the cells performing tumbling trajectories (Fig. S9A), and ii) the extremely small proportion of cells in the population that satisfy the condition $|A| \approx |\Omega|$, where the expression for the tumbling period diverges near the bifurcation. We instead expect the natural variability of the intracellular stress level s_0 (see for example Figs. S3 and S10) and of the stress dissipation timescale τ_S to play a greater role in affecting the *pdf* of steady-state stress over the population (compare for example the red and orange lines in Fig. S9D, which represent the distribution of the stress generated in two populations with increased scavenging machinery and increased mechanical stability, respectively).

In Eq. S11, we may note the (weak) quadratic dependence ½ $(A/\Omega)^2$ of the stress on the stability parameter for $|A| < |\Omega|$, as opposed to the linear dependence on the stress dissipation timescale τ_S, on the stress spikes Δs, and on the basal stress rate c_0. Below, we consider the effect of variability in the parameters τ_S and c_0 on variability in the stress accumulation.

The intracellular stress accumulation–dissipation dynamics $ds/dt = - s\,\tau_S^{-1} + c_0$ at steady state reads $s_0 = c_0\tau_S$. To obtain an indication of the degree of variability of the intracellular baseline stress level s_0, we analyzed flow cytometry data to extract the distribution of stress levels for a population of control cells treated with the fluorescent stain CM-H2DCFDA, a general oxidative stress indicator (see cyan bars in Figs. S5, S6, S8 corresponding to the treatment 'C+S'). Therefore, in order to capture the intrinsic variability in the scavenging efficiency for a population of cells, we model the distribution of the dissipation timescale with a gamma distribution $G(\tau_S; \alpha, \beta)$, with parameters α = 3.1 and β = 28.1 by fitting the experimental profiles (up to a multiplicative constant c_0) of the distribution of intracellular stress in the control from flow cytometry experiments (Fig. S10, cyan lines). In doing so, we made the assumption that all cells have the same stress rate c_0, and that the variability expressed in the baseline stress levels s_0 originates from the variability in the dissipation timescale τ_S. Note that we do not have experimental data concerning the intrinsic variability of the parameter τ_S, since our experiments to extract the dissipation timescale were performed at the population level (fitting presented in Fig. 3B in the main text). However, this parametrization preserves the mean value of the experimental dissipation timescale, $\langle\tau_S\rangle = \alpha\beta = 87$ s. We compound the sources of variation (assuming A and τ_S as independent random variables) to derive the *pdf* of the stress M(s) after exposure to rolling at a rotation rate Ω

$$M(s) = w_U \int_{2\pi(s-\Delta s/2)/(\Delta s\,\Omega)}^{4\pi(s-\Delta s/2)/(\Delta s\,\Omega)} G(\tau_s; \alpha, \beta)\ \sum\nolimits_{\pm} F(A_{\pm}(s, \tau_s); \mu, \sigma) \left|\frac{dA_{\pm}}{ds}\right| d\tau_s \ +\ w_S\, G(s_0; \alpha_0, \beta_0), \qquad \text{Eq. S14}$$

where the term $\sum_{\pm} F(A_{\pm}(s, \tau_s); \mu, \sigma)$ sums over the two branches of the *pdf*s for the stability parameter for the cells that tumble, and the terms w_U,w_S, with $w_S = 1 - w_U$, are weighting factors for the unstable (tumbling cells) and stable solutions, respectively. The weighting factors, which measure the proportion of cells that do not tumble in a population rolling at a rotation rate Ω, are calculated with the following expression

$$w_U = \int_{-\Omega}^{+\Omega} F(A; \mu, \sigma)\, dA\ , \qquad \text{Eq. S15}$$

where $F(A; \mu, \sigma)$ is the Gaussian distribution of the stability parameter fitted to the experimental data presented in Fig. S9C. In Fig. S10 the experimental stress curves for the control, top, and bottom cells after 1 min rolling for the rotation rate $\Omega = 1$ rad s^{-1} are shown, which correspond to the experimental parameters presented in Fig. 2A. The value of the stress spike generated at each tumbling event is a free parameter of the model and was fixed to $\Delta s/s_0 = 0.40$ through a least squares fitting procedure (see Fig. 3D and ***Cell stress generated by rolling*** for details on fitting procedure). Additionally, for the subpopulation at the top we implemented a smaller dissipation timescale τ_S^T in the fit for the stress levels. This difference in the dissipation timescales between top and bottom subpopulations is based on the analysis of the bistable stress response presented in Fig. 2C upon induction with H_2O_2. The intracellular stress dynamics due to H_2O_2, similarly to the stress model capturing the accumulation–dissipation dynamics due to turbulence, can be captured by the differential equation $ds/dt = - s\,\tau_S^{-1} + c_0 + c_H$, where s is the stress level, c_0 is the baseline stress rate, and c_H is the stress rate induced by H_2O_2. At steady state, the stress level reads $s_H = \tau_S\,(c_0 + c_H)$. From the stress levels recorded in the top and the bottom subpopulations in the H_2O_2 experiments (Fig. 2C), we deduced that the timescale τ_S^T for the top subpopulation is $\tau_S^T = 0.54\ \tau_S^B$, with τ_S^B the dissipation timescale of the bottom subpopulation ($\tau_S^B = \tau_S = 87$ s). We found good agreement between the distributions of stress for the two subpopulations in the experiments and in the stochastic version of our model that we implemented to explicitly capture the high degree of single-cell variability (Fig. S10).

Supplementary Figures

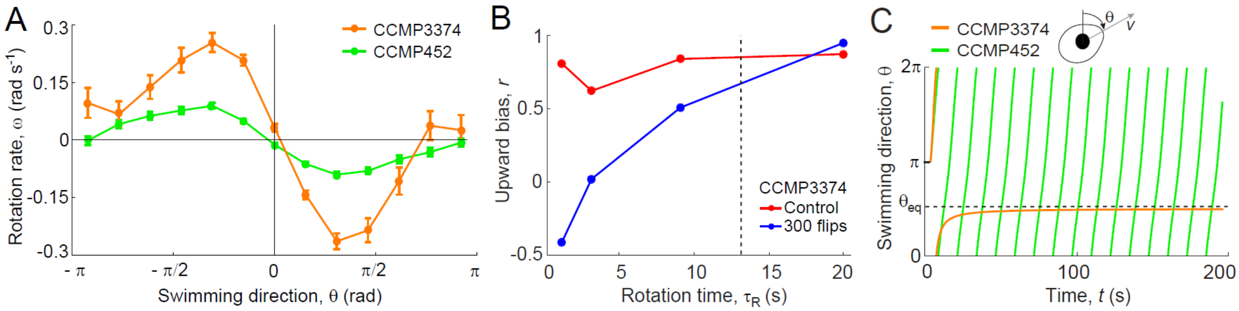

Fig. S1. The mechanical stability, together with rotation and resting timescales, determine the magnitude of splitting in *H. akashiwo* CCMP3374. (**A**) Cellular rotation rate, ω, of CCMP452 (green) and CCMP3374 cells (orange), as a function of the cell orientation to the vertical, θ, measured before flipping. Solid lines denote the arithmetic mean over all cell trajectories and error bars represent ± 1 standard error of the mean (s.e.m.). The population stability parameter (CCMP452: $A_{452} = 0.09\ s^{-1}$, and CCMP3374: $A_{3374} = 0.23\ s^{-1}$) represent the amplitude of the sinusoidal function fitted to the curve (Methods). (**B**) Upward bias, r, in CCMP3374 after $N = 300$ flips as a function of the rotation time τ_R (control cells were kept in quiescent conditions). In agreement with theoretical predictions, the rotation time τ_R in combination with the stability parameter A regulate the magnitude of the population split into two subpopulations with opposite mechanical stability (Supplementary Text). In particular, we observed that the switch of stability occurred at rotation rates for which $A_{3374}^{-1}|\Omega| > 1$ (the dashed line satisfies the condition $A_{3374}^{-1}\Omega = 1$, with $\Omega = \pi/\tau_R$). We note that the effect of reorientations on the behavioral response is greater for CCMP3374, for which the upward bias changes from $r = 0.82$ for the control to $r = -0.46$ upon treatment (the negative value indicating that a majority of cells were downward-migrating after flipping). Converting to percentages of upward-migrating cells, this corresponds to 91% of cells migrating upwards in the control and only 27% after the treatment, i.e., 64% of cells changed behavior, compared to 23% of cells changing behavior in CCMP452. (**C**) Predicted swimming direction over time for *H. akashiwo* strains with the two different stability parameters A_{452} and A_{3374} calculated in A, exposed to a solid body rotation with constant angular frequency $\Omega = 0.2$ rad s^{-1}. Cells from the CCMP3374 population, with a higher stability parameter, maintain a stable swimming direction (dashed line), $\theta_{eq} = \arcsin(A^{-1}\Omega)$, provided (as it is in this case) that the condition $|\Omega| < |A|$ is satisfied (we displayed here the case $A > 0$ for an upward-migrating cell, the angle for a downward-migrating cell is $\theta_{eq} = \pi - \arcsin(\Omega A^{-1})$). Cells from the CCMP452 population tumble and perform periodic orbits (see Eq. 2 in the main text).

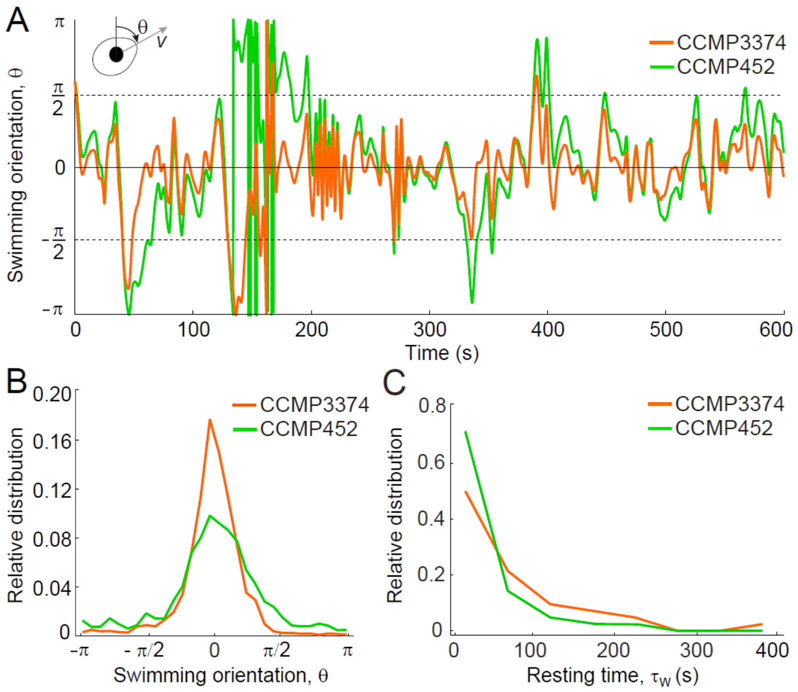

Fig. S2. The mechanical stability determines the temporal distribution of tumbling and resting times of phytoplankton under turbulence. (**A**) Time series of a cell's swimming direction relative to gravity, θ(t), calculated from Eq. 1 in the main text, where we imposed the rotation rate $\Omega(t)$, which represents the rotation rate relative to the direction of gravity of a passive sphere in a 3D isotropic turbulent flow obtained from a direct numerical simulation (Re_λ = 65, ε = 10^{-6} W kg^{-1}, ref. S15, Methods). Colored lines are obtained for two values of the stability parameter, A, corresponding to CCMP452 cells (A_{452} = 0.09 s^{-1}, green) and CCMP3374 cells (A_{3374} = 0.23 s^{-1}, orange). During times when the rotation rate is $|\Omega|A^{-1}< 1$, cells are not tumbled but will achieve an equilibrium swimming orientation (Fig. S1B), corresponding in our experiments to a resting time, τ_W, between reorientations. CCMP3374 cells, characterized by a higher mechanical stability, regain their equilibrium swimming direction more quickly than CCMP452 cells after being tumbled by turbulence. (**B**) Relative distribution of the swimming direction relative to gravity for CCMP3374 and CCMP452 cells under turbulence. Cells of CCMP3374 characterized by a higher mechanical stability spend longer period of times with an orientation close to their equilibrium orientation in the absence of fluid flow, that is the direction opposite to gravity, θ = 0. (**C**) Relative distribution of the resting time, τ_W, for CCMP452 cells (green, mean resting time: τ_{W452} = 46 s) and CCMP3374 cells (orange, mean resting time: τ_{W3374} = 104 s). The statistics for the resting times was extracted from the distribution of time periods between two tumbling events, defined as the time points with swimming direction $\theta = \pi/2$ (dashed lines in A, see Methods and Supplementary Text).

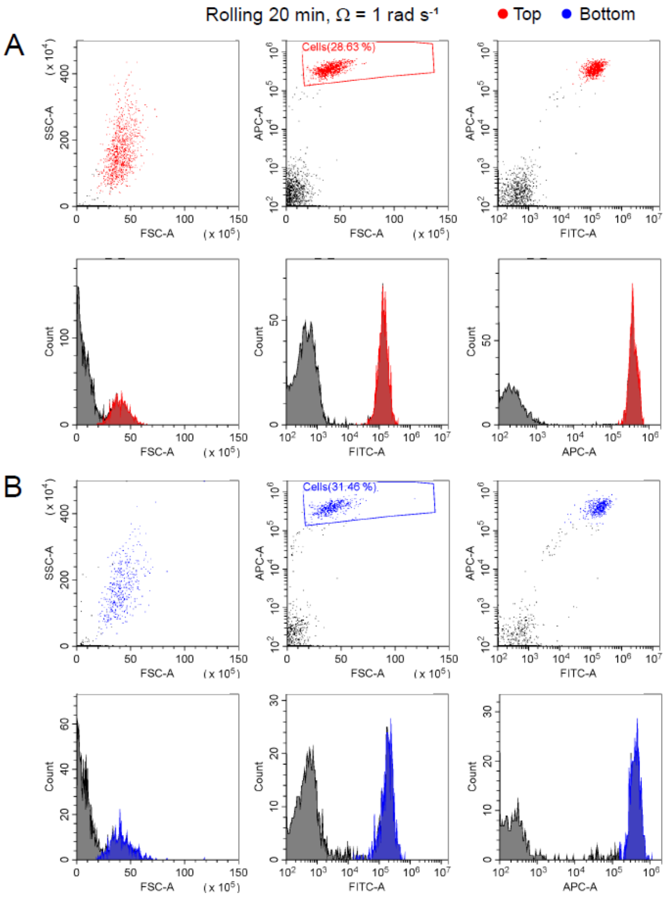

Fig. S3. Stress levels in *H. akashiwo* were higher for the bottom subpopulation. To quantify the accumulation of reactive oxygen species (ROS), cells exposed to rolling in 2 ml cylindrical vials were incubated with 10 μM CM-H_2DCFDA, a general oxidative stress marker that passively diffuses into live cells and binds to free radicals (see Methods). After a dark-incubation period (30 min), the cells were examined using a flow cytometer. Each sample was run through the flow cytometer until at least 1000 viable cells were detected. The oxidative stress levels are represented as the fluorescence readouts from the flow cytometer in the FITC-A channel, which matches the excitation/emission wavelengths of the CM-H_2DCFDA dye (Ex/Em: ~488/520 nm). The fluorescence levels were obtained for the turbulence-exposed population extracted from the top (**A**) and from the bottom (**B**) of the cylindrical vials after 20 min rolling. For A and B in the top row, scatter plots show side-scatter (SSC-A) vs. forward-scatter (FSC-A), allophycocyanin (APC-A) vs. FSC-A channels, and APC-A vs. FITC-A channels. The APC-A channel captures the autofluorescence signal from cells in the far-red region of the spectrum (763/43 nm). Each dot within scatterplots represents a single cell. To select only the values in the FITC-A channel from viable *H. akashiwo* cells for inclusion in the stress analysis (and not bacteria and other debris), we applied a gate using channels FSC-A vs. APC-A to identify cells (red and blue rectangles, % values indicate the relative proportion of cells). In the bottom row, the distributions of cells (colored) and debris (black) are shown for FSC-A, FITC-A, and APC-A. The same staining and flow cytometry protocol was used to quantify stress levels for all the rolling and the H_2O_2 experiments.

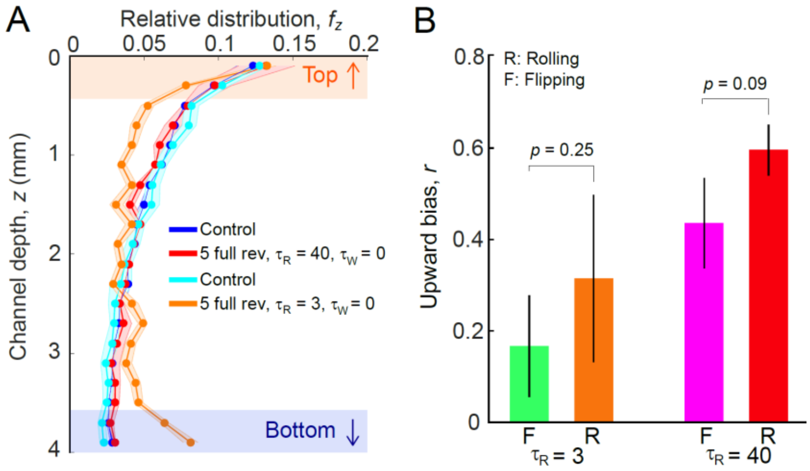

Fig. S4. Rolling produced a similar migratory response to that induced by flipping in *H. akashiwo* CCMP452. (**A**) The relative distribution of cells in the chamber, f_Z, was analyzed before (quiescent controls, cyan and blue) and after exposure to continuous rotation (rolling) of the chamber (5 full revolutions with τ_W = 0 s) at two different rotation rates (Ω = 1 rad s^{-1}, equivalent to a rotation time τ_R = 3 s, orange line; Ω = 0.08 rad s^{-1}, equivalent to a rotation time τ_R = 40 s, red line) following a period of 30 min in which the cell distribution was allowed to equilibrate. Shaded regions in orange and blue represent the top (↑) and the bottom (↓) 400 µm of the chamber, where the concentration of the cells was quantified for the calculation of the upward bias. The upward bias index, $r = (f_{\uparrow} - f_{\downarrow})/(f_{\uparrow} + f_{\downarrow})$, measures the relative proportion of up-swimming ($f_{\uparrow}$) and down-swimming ($f_{\downarrow}$) cells. (**B**) The upward bias obtained in the continuous rolling experiments (R) compared to the flipping experiments (F; N = 10 flips, with the corresponding rotation times and zero resting time, τ_W = 0 s). No difference in the upward bias was detected between rolling and flipping (two-sample t-tests; τ_R = 3 s rotation time, t_7 = 1.26, p = 0.25; τ_R = 40 s rotation time, t_5 = 2.09, p = 0.09).

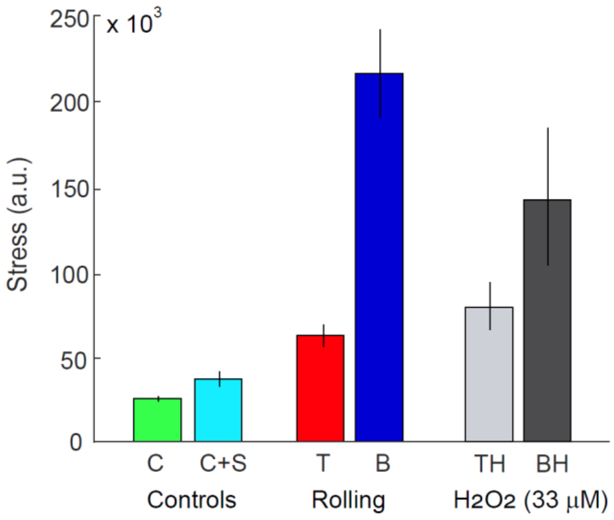

Fig S5. Bistability in oxidative stress mediates vertical migration of *H. akashiwo* CCMP3374. Oxidative stress level caused by intracellular ROS accumulation after exposure to 20 min rolling (Ω = 1 rad s^{-1}) and by exposure to exogenous H_2O_2 (concentration C = 33 µM) in *H. akashiwo* CCMP3374. Bars show the oxidative stress levels for the top (red, 'T' for rolling; light gray, 'TH' for H_2O_2) and the bottom (blue, 'B' for rolling; dark gray, 'BH' for H_2O_2) subpopulations. Also shown are the baseline fluorescence signal of untreated control cells (green, 'C') and of control cells treated with the fluorescent stain CM-H2DCFDA, a general oxidative stress indicator (cyan, 'C+S'). Stress levels were computed from flow cytometric measurements (mean ± s.d. of three replicates). Similarly to the strain CCMP452 (Fig. 2C), cells that switch to downward-swimming behavior show elevated stress levels ('B' and 'BH'), while cells that continue to swim upward ('T' and 'TH') show stress levels closer to those of the controls.

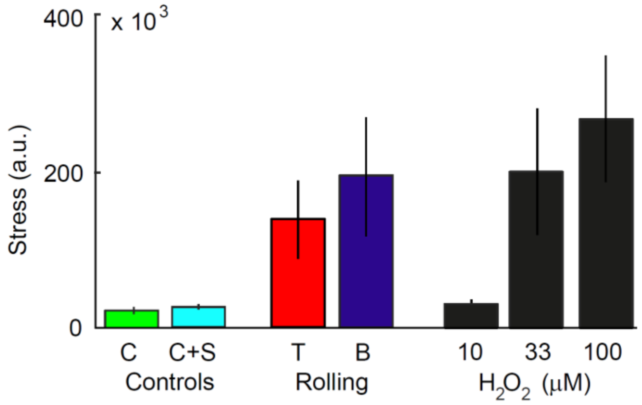

Fig. S6. Rolling produced a similar stress response to that induced by exposure to hydrogen peroxide in *H. akashiwo* CCMP452. Oxidative stress level caused by intracellular ROS accumulation for cells exposed to 20 min rolling ($\Omega = 1$ rad s^{-1}) (red and blue bars, denoting the top 'T' and bottom 'B' subpopulations, respectively) or caused by exposure to different concentrations of exogenous hydrogen peroxide, H_2O_2 (black bars). Also shown are the baseline fluorescence signal of untreated control cells (green, 'C') and of control cells treated with the fluorescent stain CM-H_2DCFDA, a general oxidative stress indicator (cyan, 'C+S'). Stress levels were computed from flow cytometric measurements (mean ± s.d. of three replicates).

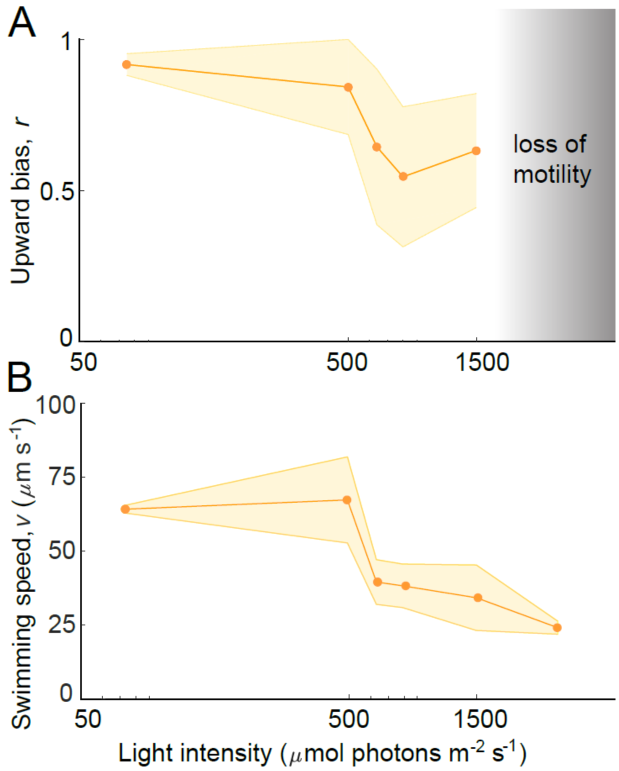

Fig. S7. Upward bias (A) and swimming speed (B) of a population of *H. akashiwo* CCMP452 as a function of light intensity. We observed a threshold response of the upward bias with increasing light intensity, and a loss of motility (gray shaded region in A) for cells exposed to full spectrum irradiance higher than 650 μmol photons m^{-2} s^{-1}. An almost complete loss of motility was observed for light intensities higher than 1500 μmol photons m^{-2} s^{-1}, where the values of swimming speed (v = 22 μm s^{-1}) were comparable to the sinking speed predicted by the Stokes' law, $v_s = 2/9(\rho_p - \rho_f)g\mu^{-1}R^2$, which for *H. akashiwo* is v_s = 6 μm s^{-1}, where $(\rho_p - \rho_f)$ = 50 kg m^{-3} is the excess density, g = 9.8 m s^{-2} is the gravitational acceleration, μ = 10^{-3} Pa s is the dynamic viscosity of seawater, and R = 7 μm is the equivalent radius of the cell (*S1*, *S9*, *S10*). The minimum value of light intensity (75 μmol photons m^{-2} s^{-1}) corresponds to the intensity used for culturing the population (Methods). Points represent the mean of two replicates and the shaded region is ±1 s.d.

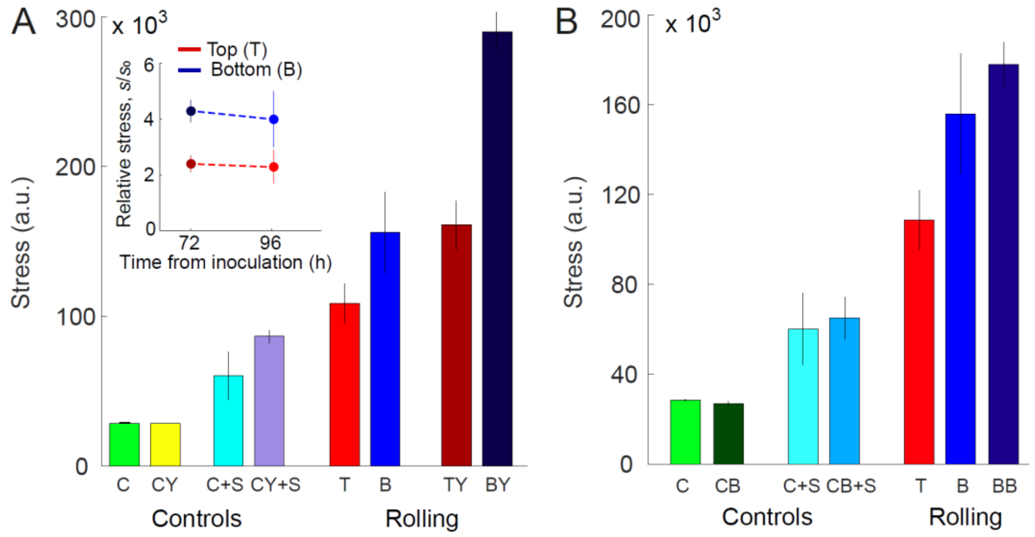

Fig. S8. Stress accumulation after exposure to rolling in *Heterosigma akashiwo* CCMP452 is regulated by the growth phase. (**A**) Oxidative stress level caused by intracellular ROS accumulation after exposure to 20 min rolling ($\Omega = 1$ rad s^{-1}). Bars show the oxidative stress levels after rolling for the subpopulations from the top tested 96 h ('T') and 72 h after inoculation ('TY'), and from the bottom after 96 h ('B') and after 72 h ('BY'). Also shown are the baseline fluorescence signal of untreated control cells 96 h ('C') and 72 h after inoculation ('CY'), of control cells treated with the fluorescent stain CM-H2DCFDA after 96 h ('C+S') and after 72 h ('CY+S'). The inset shows the stress accumulated after exposure to rolling for the top (red dots) and bottom (blue dots) subpopulations for cells 72 h and 96 h after inoculation relative to the respective controls. Specifically, we calculated the quantities $h_{BY} = (s_{BY} - s_{CY})/(s_{CY+S} - s_{CY}) = 4.3 \pm 0.4$; $h_{TY} = 2.4 \pm 0.3$; $h_B = 4.0 \pm 1.0$; $h_T = 2.3 \pm 0.6$, which represent the relative increase in ROS for the bottom and top subpopulations upon rolling for cultures after 72 h (BY, TY) and 96 h (B, T), after subtraction of the characteristic autofluorescence of *H. akashiwo* over the green portion of the spectrum. Cells 72 h after inoculation present a higher baseline ROS production rate compared to cells after 96 h (compare bars C+S and CY+S), though the relative increase in the ROS upon rolling for both the top and bottom subpopulations is comparable between the two growth phases. Stress levels were computed from flow cytometric measurements (mean ± s.d. of at least two replicates). (**B**) Initially downward-migrating *H. akashiwo* CCMP452 cells extracted from the bottom in the control experiment, likely freshly divided cells, show increased stress after exposure to rolling. According to literature (S20), cell division in *H. akashiwo* occurs as a synchronous event during the cell cycle when cells are subjected to light-dark regimes, such as in our growth conditions (see Methods in the main text). The bottom-dwelling cells in the control could be freshly divided cells that naturally exhibit a downward swimming pattern because of their symmetric morphology and the fact that they are top-heavy (*S1*). Oxidative stress level caused by intracellular ROS accumulation after exposure to 20 min rolling ($\Omega = 1$ rad s^{-1}). Bars show the oxidative stress levels after rolling for the top (red, 'T') and the bottom (blue, 'B') subpopulations, and for cells that were downward migrating in the control and were then found in the bottom subpopulation after being exposed to rolling (dark blue, 'BB'). Also shown are the baseline fluorescence signal of untreated control cells (green, 'C'), of untreated control cells from the bottom (dark green, 'CB'), of control cells treated with the fluorescent stain CM-H2DCFDA, a general oxidative stress indicator (cyan, 'C+S') and of control cells from the bottom treated with the stain (dark cyan, 'CB+S'). Stress levels were computed from flow cytometric measurements (mean ± s.d. of at least two replicates).

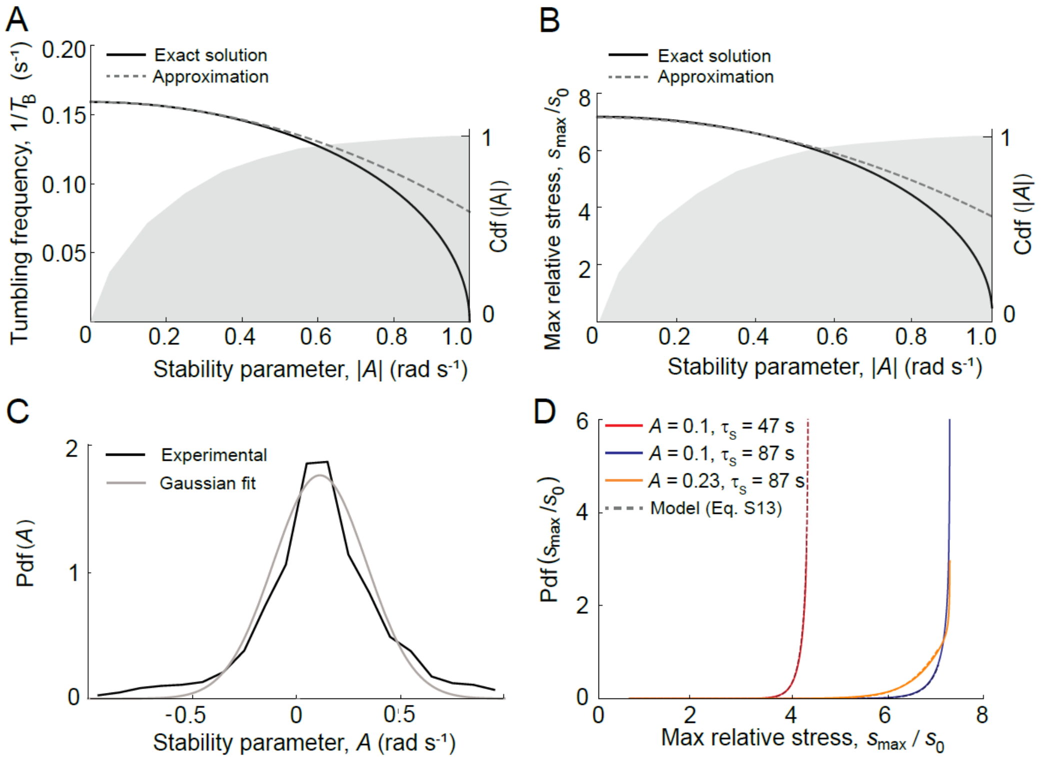

Fig. S9. Stochastic analysis of tumbling rate and stress accumulation for cells of *H. akashiwo* under continuous rolling. (**A**) Tumbling frequency, which is the inverse of the tumbling period T_B, for cells subjected to rolling at a rotation rate $\Omega = 1$ rad s^{-1}, decreases as a function of the stability parameter A for cells within a population where $|\Omega| > |A|$. The black line is the exact solution $T_B^{-1} = (2\pi)^{-1}(\Omega^2 - A^2)^{1/2}$, whereas the gray dotted line is the approximation presented in Eq. S10. The gray shaded region represents the cumulative distribution function for the experimental distribution of the stability parameter presented in Fig. 1E. This analysis shows very good agreement (<1% error) between our approximation and the exact solution for the vast majority (95%) of the cells in the population. (**B**) Maximum relative stress at steady state accumulated for cells after exposure to rolling as in panel A, as a function of the stability parameter A. The black line is the exact solution $s_{max}/s_0 = \Delta s/s_0 \left(1 - e^{-T_B \tau_S^{-1}}\right)^{-1} + 1$, whereas the gray dotted line is the approximation presented in Eq. S11. The gray shaded region represents the cumulative distribution function of the stability parameter as in panel A. This analysis shows very good agreement (<1% error) between our approximation and the exact solution for the vast majority (95%) of the cells in the population. (**C**) Relative distribution of the stability parameter A (as in Fig. 1E, red curve). The black line is the experimental distribution, whereas the gray line is a fit through a Gaussian distribution with parameters $\mu = 0.10$ rad s^{-1} and $\sigma = 0.21$ rad s^{-1} for the mean and standard deviation, respectively. (**D**) Probability density function for the maximum relative stress accumulated upon exposure to rolling at a rotation rate $\Omega = 1$ rad s^{-1}. Colored lines depict the exact numerical distribution for different values of the population parameters of the mechanical stability A and of the stress dissipation timescale τ_S (blue and red lines would correspond to top and bottom subpopulations of CCMP452, respectively, and the orange line would correspond to CCMP3374, with parameters $\mu = 0.23$ rad s^{-1} and $\sigma = 0.21$ rad s^{-1} and assuming a dissipation timescale $\tau_S = 87$ s as in CCMP452). Corresponding dotted lines are computed using the approximated expression $H(s)$ provided in Eq. S13. At the fast rotation rate considered ($\Omega = 1$ rad s^{-1}), the greatest impact on the accumulated stress distribution results from the dissipation timescale, compared to a relatively small effect of the stability parameter.

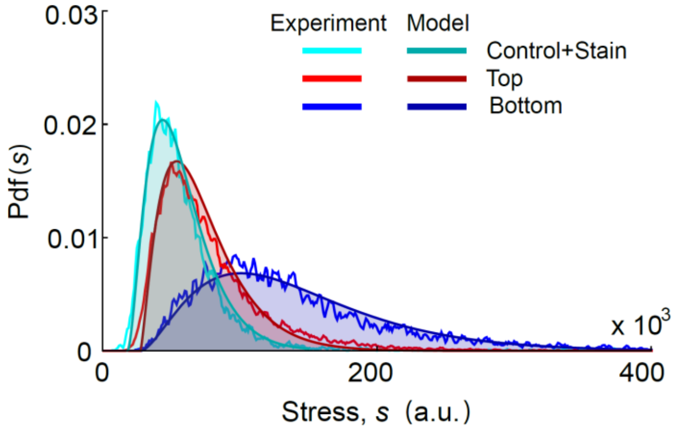

Fig. S10. The distribution of accumulated stress in the two subpopulations of *H. akashiwo* CCMP452 upon rolling is captured by the stochastic model of stress dynamics. The lighter colored lines represent the *pdf*s of accumulated stress for cells exposed to 1 min rolling at a rotation rate Ω = 1 rad s^{-1} (data taken from Fig. 2A). Darker colored lines correspond to the solution of the stress model of Eq. S14. The gamma distribution $G(\tau_S;\, \alpha,\, \beta)$ for the bottom subpopulation (blue lines) has parameters α = 3.1 and β = 28.1 (τ_S^B = 87 s), obtained by fitting the experimental profile of the control where cells were treated with the fluorescent stain CM-H2DCFDA (light cyan). The gamma distribution $G(\tau_S^T;\, \alpha,\, \beta)$ for the top subpopulation (red lines) has parameters α = 2.4 and β = 19.6 to match the lower values of the timescale τ_S^T = 0.54 τ_S^B = 47 s. The value of the stress spike generated at each tumbling event is a fitting parameter of the model and was fixed to $\Delta s/s_0$ = 0.4 (see Fig. 3D and Supplementary Text ***Cell stress generated by rolling*** for further details on the fitting procedure of the parameter Δs).

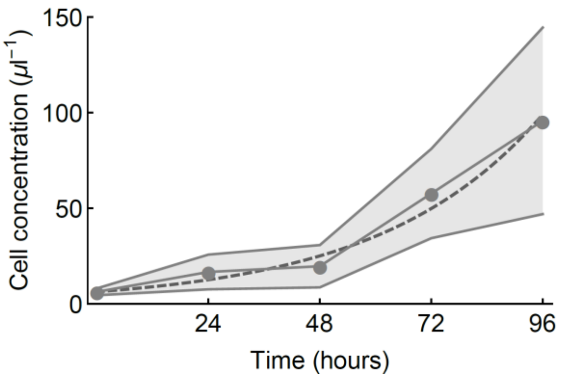

Fig. S11. The growth curve of *H. akashiwo* CCMP452. To obtain the growth curve in the control, we sampled cells every 24 h, grown under the same conditions as the cells for the top and bottom subpopulations (Fig. 4B in the main text). The intrinsic growth rate, *g*, was quantified by fitting an exponential function (dashed curve; $g = 0.69 \pm 0.06$ day^{-1}). This value for the growth rate in the control cells is not different from the growth rate of the top subpopulation after exposure to turbulence ($g_{\uparrow} = 0.74 \pm 0.02$ day^{-1}).

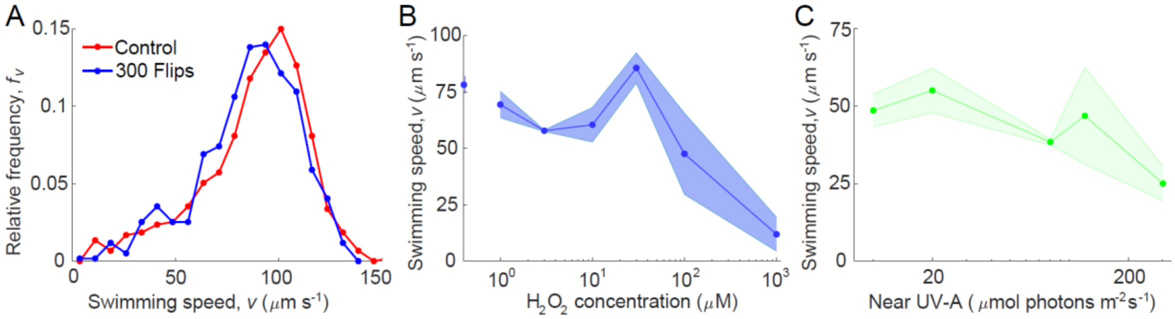

Fig. S12. Modulation of swimming speed for *H. akashiwo* CCMP452 after exposure to different stressors. (**A**) No difference in the distribution of swimming speeds was detected between a population of cells before (control, red curve, v = 88 ± 33 μm s^{-1}) and after (blue curve, v = 86 ± 29 μm s^{-1}) exposure to N = 300 flips at the highest rotation rate used in our experiments (Ω = 3.14 rad s^{-1} , which corresponds to a rotation time τ_R = 1 s), with zero resting time between flips (τ_W = 0 s). (**B**) Swimming speed after exposure of a population of cells to different concentrations of H_2O_2. The blue point on the *y*-axis indicates the control. A consistent drop in motility was observed at a concentration of 100 μM H_2O_2, and a complete loss of motility occurred at a concentration of 1 mM H_2O_2, where the detected swimming speed, v = 11 μm s^{-1}, was comparable to the sinking speed predicted by the Stokes' law, $v_s = 2/9(\rho_p - \rho_f)g\mu^{-1}R^2$, which for *H. akashiwo* is v_s = 6 μm s^{-1}, where ($\rho_p - \rho_f$) = 50 kg m^{-3} is the excess density, g = 9.8 m s^{-2} is the gravitational acceleration, μ = 10^{-3} Pa s is the dynamic viscosity of seawater, and R = 7 μm is the equivalent radius of the cell (*S1*, *S9*, *S10*). Points represent the mean of three replicates and the shaded region is ± 1 s.d. (**C**) Swimming speed after exposure of a population of cells to different UV-A intensities. Cells maintained normal motility after exposure up to an intensity of 120 μmol photons m^{-2} s^{-1} UV-A. A drop in motility was detected at a photon flux density of 300 μmol photons m^{-2} s^{-1} UV-A. Points represent the mean of three replicates and the shaded region is ± 1 s.d.

Table S1 | Multiple comparisons analysis (Tukey's HSD test) of the upward bias of *H. akashiwo* CCMP452 among a population that was flipped, a population that was flipped after having been cultured in the presence of a scavenger of reactive oxygen species (potassium iodide), and a control population (without flipping and no potassium iodide). Control = quiescent control; Scavenger = a population grown in f/2 medium with potassium iodide at a concentration of 100 μM, flipped 100 times $\tau_R = 3$ s, $\tau_W = 0$ s; Flipped = a population grown in f/2 medium, flipped 100 times ($\tau_R = 3$ s, $\tau_W = 0$ s).

Treatments compared	Mean difference (95% confidence interval)	*p*-value
Control, Scavenger	0.202 (-0.03, 0.434)	0.085
Control, Flipped	0.459 (0.242, 0.676)	0.001
Scavenger, Flipped	0.257 (0.04, 0.474)	0.024

Table S2 | Multiple comparisons analysis (Tukey's HSD test) of the photosynthetic quantum yield *H. akashiwo* CCMP452 among subpopulations that were collected from the top and bottom after rolling, and a control population (without rolling). Control = quiescent control; Top = subpopulation harvested from the top of the chamber, 5 min rolling time (Ω = 1 rad s^{-1}); Bottom = subpopulation harvested from the bottom of the chamber, 5 min rolling time (Ω = 1 rad s^{-1}).

Treatments compared	Mean difference (95% confidence interval)	*p*-value
Control, Top	0.01 (-0.03, 0.05)	0.799
Control, Bottom	0.09 (0.05, 0.13)	<0.001
Top, Bottom	0.08 (0.04, 0.12)	<0.001

Supplementary References

S1. A. Sengupta, F. Carrara, R. Stocker, Phytoplankton can actively diversify their migration strategy in response to turbulence cues, *Nature* **543**, 555-558 (2017).

S2. A. Thorpe, *An introduction to ocean turbulence* (Cambridge Univ. Press, Cambridge, MA, 2007).

S3. H. L. Fuchs, G. P. Gerbi, Seascape-level variation in turbulence- and wave-generated hydrodynamic signals experienced by phytoplankton, *Prog. Ocean.* ***141***, 109-129 (2016).

S4. K. W. Foster, R. D. Smyth, Light antennas in phototactic algae, *Microbiol. Rev.* ***44***, 572–630 (1980).

S5. N. Oulette, H. Xu, E. Bodenschatz, A quantitative study of three-dimensional Lagrangian particle tracking algorithms, *Exp. Fluids* ***40***, 301-313 (2006).

S6. T. Fenchel, How dinoflagellates swim, *Protist* ***152***, 329-338 (2002).

S7. A. M. Roberts, Geotaxis in motile microorganisms, *J. Exp. Biol.* ***53***, 687–699 (1970).

S8. H. M. Shapiro, *Practical Flow Cytometry*, (Wiley & Sons, New York, NY, 2003).

S9. Y. Hara, M. Chihara, Morphology, ultrastructure and taxonomy of the raphidophycean alga *Heterosigma akashiwo*, *Bot. Mag.* ***100***, 151-163 (1987).

S10. M. Wada, A. Miyazaki, T. Fujii, On the mechanisms of diurnal vertical migration behavior of *Heterosigma akashiwo* (Raphidophyceae), *Plant Cell. Physiol.* ***26***, 431-436 (1985).

S11. K. Mukai, Y. Shimasaki, X. Qiu, Y. Kato-Unoki, K. Chen, M. R. M. Khanam, Y. Ohima, Effects of light and hydrogen peroxide on gene expression of newly identified antioxidant enzymes in the harmful algal bloom species *Chattonella marina*, *Eur. J. Phyc.* ***54***, 393-403 (2019).

S12. C. L. Dupont, T. J. Goepfert, P. Lo, L. Wei, B. A. Ahner, Diurnal cycling of glutathione in marine phytoplankton: Field and culture studies, *Limnol. Oceanogr.* **49**, 991-996 (2004).

S13. T. J. Pedley, J. O. Kessler, Hydrodynamic phenomena in suspensions of swimming microorganisms, *Ann. Rev. Fluid Mech.* **24**, 313-358 (1992).

S14. S. B. Pope, *Turbulent flows* (Cambridge Univ. Press, Cambridge, MA, 2000)

S15. W. M. Durham, E. Climent, M. Barry, F. De Lillo, G. Boffetta, M. Cencini, R. Stocker, Turbulence drives microscale patches of motile phytoplankton, *Nat. Commun.* ***4***, 2148 (2013).

S16. R. E. Brier, C. C. Lalescu, D. Waas, M. Wilczek, M. G. Mazza, Emergence of phytoplankton patchiness at small scales in mild turbulence, *Proc. Natl. Acad. Sci. U.S.A.* **115**, 12112-12117 (2018).

S17. M. J. Zirbel, F. Veron, M. I. Latz, The reversible effect of flow on the morphology of *Ceratocorys horrida* (Peridiniales, Dinophyta), *J. Phycol.* **36**, 46-58 (2000).

S18. E. Berdalet, F. Peters, V. L. Koumandou, C. Roldan, O. Gudayol, M. Estrada, Species-specific physiological response of dinoflagellates to quantified small-scale turbulence, *J. Phycol.* **43**, 965-977 (2007).

S19. A. Amato, G. Dell'Aquila, F. Musacchia, R. Annunziata, A. Ugarte, N. Maillet, A. Carbone, M. Ribera d'Alcalà, R. Sanges, D. Iudicone, M. I. Ferrante, Marine diatoms change their gene expression profile when exposed to microscale turbulence under nutrient replete conditions, *Sci. Rep.* **7**, 3826 (2017).

S20. E. Satoh, M. M. Watanabe, T. Fujii, Photoperiodic regulation of cell division and chloroplast replication in Heterosigma akashiwo, *Plant Cell Physiol.* **28**, 1093-1099 (1987).
